# Meta-analysis of laparoscopic radical hysterectomy, excluding robotic assisted versus open radical hysterectomy for early stage cervical cancer

**DOI:** 10.1038/s41598-023-27430-9

**Published:** 2023-01-06

**Authors:** Greg Marchand, Ahmed Taher Masoud, Ahmed Abdelsattar, Alexa King, Hollie Ulibarri, Julia Parise, Amanda Arroyo, Catherine Coriell, Sydnee Goetz, Carmen Moir, Atley Moberly, Malini Govindan

**Affiliations:** 1 Marchand Institute for Minimally Invasive Surgery, 10238 E. Hampton, Ste. 212, Mesa, AZ 85209 USA; 2 Faculty of Medicine, Fayoum University, Fayoum, Egypt

**Keywords:** Cervical cancer, Outcomes research

## Abstract

Recent evidence has shown an increase in recurrence and a decrease in overall survival in patients treated with laparoscopic radical hysterectomy (LRH) and robotic assisted radical hysterectomy (RRH) open techniques (ORH). In addition, several high quality trials were recently published regarding the laparoscopic treatment of early stage cervical cancer. We sought out to reassess the recurrence rates, overall survival, complications and outcomes associated with laparoscopic radical hysterectomy (LRH) techniques against open techniques (ORH) when robotic assisted techniques were excluded. We searched PubMed, Medline, Cochrane CENTRAL, SCOPUS, ClinicalTrials.Gov and Web of Science for relevant clinical trials and observational studies. We included all studies that compared with early stage cervical cancer receiving LRH compared with ORH. We included randomized clinical trials, prospective cohort, and retrospective cohort trials. We included studies that included LRH and RRH as long as data was available to separate the two arms. We excluded studies that combined LRH and RRH without supplying data to differentiate. Of 1244 total studies, we used a manual three step screening process. Sixty studies ultimately met our criteria. We performed this review in accordance with PRISMA guidelines. We analyzed continuous data using mean difference (MD) and a 95% confidence interval (CI), while dichotomous data were analyzed using odds ratio (OR) and a 95% CI. Review Manager and Endnote software were utilized in the synthesis. We found that when excluding RRH, the was no significant difference regarding 5-year overall Survival (OR = 1.24 [0.94, 1.64], (*P* = 0.12), disease free survival (OR = 1.00 [0.80, 1.26], (*P* = 0.98), recurrence (OR = 1.01 [0.81, 1.25], (*P* = 0.95), or intraoperative complications (OR = 1.38 [0.94, 2.04], (*P* = 0.10). LRH was statistically better than ORH in terms of estimated blood loss (MD = − 325.55 [− 386.16, − 264.94] (*P* < 0.001), blood transfusion rate (OR = 0.28 [0.14, 0.55], (*P* = 0.002), postoperative complication rate (OR = 0.70 [0.55, 0.90], (*P* = 0.005), and length of hospital stay (MD = − 3.64[− 4.27, − 3.01], (*P* < 0.001). ORH was superior in terms of operating time (MD = 20.48 [8.62, 32.35], (*P* = 0.007) and number of resected lymph nodes (MD = − 2.80 [− 4.35, − 1.24], (*P* = 0.004). The previously seen increase recurrence and decrease in survival is not seen in LRH when robotic assisted techniques are included and all new high quality is considered. LRH is also associated with a significantly shorter hospital stay, less blood loss and lower complication rate.

Prospero Prospective Registration Number: CRD42022267138.

## Introduction

Cervical cancer is the third most common malignancy and the second most common cause of death from cancer among women in the USA^1^. The incidence and mortality of cervical cancer have a large geographic variation as it is more common and more fatal in developing countries^2^. Cervical cancer is classified into early-stage and advanced-stage cancer^3^. Although staged clinically, the surgical treatment of early-stage cervical treatment is critical for optimizing patient survival^4,5^. Radical hysterectomy with pelvic lymphadenectomy is a commonly performed procedure for the treatment of cervical cancer^6^. Patients who are found to be candidates for surgical intervention may see 5-year survival rate increased to as high as 87%^7^. Abdominal, laparoscopic, robotic-assisted laparoscopic and vaginal approaches to radical hysterectomy have all been described and performed by many authors^8^. Secondary to the risk of spreading cancers, several authors have stated that gynecologic oncology has been slower to adopt minimally invasive techniques than other specialties, sometimes reserving these techniques for only risk reducing procedures^9^. The first laparoscopic radical hysterectomy was performed in 1993^10^ and since that time the minimally invasive surgery (both laparoscopic and robotic-assisted laparoscopic) have been increasingly used.^11,12^. Over the last two decades, many studies have compared the survival outcomes and operative morbidity of the minimally invasive and open surgery for the management of cervical cancer^13^. Open radical hysterectomy shows significant morbidity including bladder dysfunction, blood loss, and complications of blood transfusion^14^. Many retrospective studies^15–19^ have shown that laparoscopic surgery causes perioperative complications less than open surgery. Complicating this, a recent randomized clinical multicenter trial proved that minimally invasive surgery was accompanied by a high rate of recurrence and a worse disease-free survival rate than open surgery^20^. In addition, a recent retrospective study that included 2461 patients with cervical cancer showed that minimally invasive surgery is associated with a higher risk of death than open surgery^21^. The culmination of these results was a change in the National Comprehensive Cancer Network (NCCN) guidelines with regards to minimally invasive radical hysterectomy^22^. The current NCCN guidelines and European Society of Gynaecological Oncology (ESGO) guidelines recommended that the standard surgical approach for management of early-stage cervical cancer is abdominal radical hysterectomy^23^.

In addition to this recent evidence against minimally invasive radical hysterectomy techniques, several new high quality trials regarding LRH and RRH have been published. For this reason, we were motivated to investigate LRH versus ORH while excluding robotic assisted techniques. We conducted this meta-analysis with late breaking, high quality data to again compare the efficacy and safety of laparoscopic radical hysterectomy with that of open radical hysterectomy for the management of cervical cancer. We have meticulously excluded all robotic assisted cases, and attempted to obtain data to use as many high quality trials as possible, while excluding robotic assisted cases.

## Method

This meta-analysis was performed following the Preferred Reporting Items for Systematic Reviews and Meta-Analyses (PRISMA)^24^ and the guidelines reported in the Cochrane Handbook for Systematic Reviews of Interventions^25^.

### Eligibility criteria

The inclusion criteria included studies that met all of the following:

1. Population: women with early stage cervical cancer
2. Intervention: Laparoscopic radical hysterectomy (LRH)
3. Comparator: Open radical hysterectomy (ORH)
4. Included Study Designs: randomized clinical trials, prospective cohort, and retrospective cohort studies.

And included at least one of our primary or secondary outcomes.

Primary outcomes included:

(1.) Operative time, (2.) Estimated blood loss, (3.) Rate of intraoperative complications, (4.) Rate of postoperative complications, (5.) Rate of blood transfusions.

Secondary outcomes included:

(1.) Rate of recurrence, (2.) Postoperative or intraoperative mortality (defined as within 90 days of surgery), (3.) Five-year survival rate, (4.) Disease-free survival rate, (5.) Number of resected lymph nodes.

We made all efforts to obtain all data possible from all included studies in order to include as many high quality trials as possible, but ultimately excluded those trials that failed our inclusion critieria because they did not publish, or despite our best efforts we could not obtain the necessary data to differentiate which cases included robotic assistance and which did not. We excluded all secondary works, such as meta-analyses and reviews, all animal studies, conference abstracts, and studies with incomplete reported data.

### Search and study selection

We searched PubMed, Medline, Scopus, Web of Science, ClinicalTrials.Gov and Cochrane CENTRAL databases from database inception until January 2nd 2022 for articles that matched our inclusion criteria.

We used the following search strategy in our search: (laparoscopic OR laparoscopy) AND (open OR abdominal) AND ((cervical cancer) OR (cancer cervix)) AND (hysterectomy). We screened the included articles in three steps. The first step implied importing the results from electronic databases to a Microsoft Excel^26^ sheet using EndNote Software^27^. The second step included a manual title and abstract screening of the articles imported to the Excel sheet. The third step was the full-text screening of the included citations from step 2. Additionally, we manually searched the references of the included papers for possible missed studies. Two researchers separately performed the literature search and eligibility match. Disagreements in the included studies was reached by consensus. A third member of the research team was assigned to settle disputes if consensus could not be reached on any study's eligibility, but was ultimately never needed.

### Data collection

We collected three categories of data from each included study: the first category is the baseline and demographic characteristics of the included participants, such as the author, year, country, sample size, age, BMI, included stages, follow up period, time period, adenocarcinoma, squamous cell carcinoma, and positive lymph nodes. The second category included the outcomes of analysis, mainly: Operative time, estimated blood loss, Intraoperative complication, Postoperative complication, Blood transfusion rate, Recurrence, mortality, Five-year survival rate, Disease-free survival, and Resected lymph nodes. The third category included data for risk of bias assessment. The process of data collection was done using Microsoft Excel^26^.

### Risk of bias assessment

We used the quality assessment tools from the National Heart, Lung, and Blood Institute (NHLB) to assess the risk of bias of observational studies^28^. We followed The Grading of Recommendations Assessment, Development and Evaluation (GRADE) Guidelines in assessing the quality of this study. We assessed the risk of bias of included trials using Cochrane’s risk of bias tool^29^. The tool assesses proper randomization of patients, allocation concealment, and adequate blinding through seven domains. Each domain was judged to be either “low”, “unclear”, or “high” risk of bias. The details of the GRADE assessment of each outcome can be found in Supplementary Table S1.

### Analysis

We performed the meta-analysis of this study using Review Manager Software^27^. Our study included continuous and dichotomous outcomes. We analyzed continuous data using mean difference (MD) and 95% confidence interval (CI), while dichotomous data were analyzed using odds ratio (OR) and 95% CI. The fixed-effects model was used when data were homogeneous, while heterogeneous data were analyzed under a random-effects model. To measure the presence of inconsistency among the studies, we used the I^2^ and the p-value of the Chi-square tests^30^. Values of *P* < 0.1 or I^2^ > 50% were significant indicators of the presence of heterogeneity. We tried to solve the inconsistency of heterogeneous outcomes using Cochrane’s leave-one-out method^30^.

### Ethics approval and consent to participate

This Manuscript has been reviewed by the institutional IRB board at Marchand Institute and was found to be exempt from IRB review. (January 2022). Data used was exempt from consent to participate or publish secondary to the nature of the study being a systematic review, retrospectively looking at previously published data.

### Commitment to diversity

The Marchand Institute remains committed to diversity and tolerance in its research, and actively maintains a workplace free of racism and sexism. Greater than half of the authors for this study are female and many represent diverse backgrounds and under-represented ethnic groups.

## Results

### Summary of included studies

**Figure 1:**
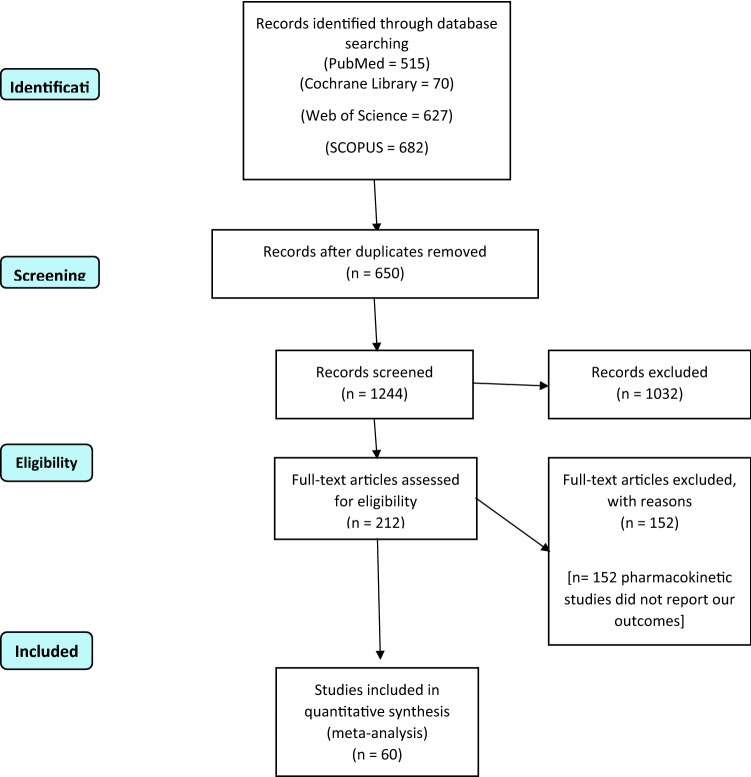
PRISMA flow diagram of our literature search.

The literature search results are illustrated in the PRISMA flow diagram in Fig. 1. We included sixty studies that met our eligibility criteria from the different databases^11,15,16,31–87^. We analyzed 42,994 patients with cervical cancer in different stages according to FIGO staging (2009 edition)^3^. A total of 15,995 patients were allocated to the laparoscopic group, while 26,999 patients were allocated to the laparotomy group. The mean age of patients in the laparoscopic group and laparotomy group was 46.5 and 46.7 years, respectively. A summary of the included studies, the demographic data of patients, the included stages, and follow-up duration are described in detail in Tables 1 and 2.

**Table 1:** Summary of the included studies.

Study ID	Country	Study design	Included stages	Follow up (mo), (median)	Time period	Sample size, n	
						LH	OH
Abu-Rustum 2003	USA	Retrospective cohort	IA1, 2–IB1	NR	2000–2002	19	195
Anagnostopoulos 2017	UK	Retrospective cohort	IA2–IIA1–IB1	36	2011–2013	36	36
Anchora 2019	Italy	Retrospective cohort	IA1	49	2016	206	217
Bogani 2014	Italy	Prospective cohort	IA2, IIA, IB2, IIB	36	2004–2011	65	65
Bogani 2020	Italy	Retrospective matched	IB1–IIA	59	2013–2014	70	35
Campos 2013	Brazil	Clinical trial	IA2–IB	NR	1999–2004	16	14
Campos 2021	Brazil	Clinical trial	IA2–IB–IIA	60	1990–2004	16	14
Chen 2014	Taiwan	Retrospective cohort	IA–IIB	NR	2005–2013	32	44
Chen 2019	China	Retrospective cohort	IB1	50	2010–2018	129	196
Chen 2020	China	Retrospective cohort	IB1	36	2004–2016	963	1634
Chen 2021	Italy	Retrospective matched	IB1	42	2008–2018	87	174
Corrado 2018	Italy	Retrospective cohort	IB1	82	2001–2016	152	101
Estape 2009	USA	Retrospective cohort	IA2, IB1, IB2	45	2006–2008	17	14
Ditto 2015	Italy	Retrospective cohort	IA2–IB1	40	2002–2013	60	60
Frumovitz 2007	USA	Retrospective cohort	IA1, 2–IB1, 2	13	2004–2006	35	54
Ghezzi 2007	Italy	Retrospective cohort	IA2, IIA, IB1, IB2	58	2004–2007	50	48
Gil-Moreno 2018	Spain	Prospective cohort	IA2, IIA2–IB1, IIB	112	1999–2016	90	76
Gortchev 2012	Bulgaria	Retrospective cohort	IB1	26	2006–2010	46	175
Guangyi 2007	China	Retrospective cohort	IB–IIA	26	1998–2005	90	35
Guo 2018	China	Retrospective cohort	IA–IIA	39	2000–2003	412	139
He 2020	China	Retrospective matched	IA1–IA2–IB1	42	2004–2016	739	739
Kanao 2019	Japan	Retrospective cohort	IB1–IIB	30	2014–2017	80	83
Kim 2018	Korea	Retrospective	NR	NR	2011–2014	3100	3235
Kim 2019	Korea	Retrospective cohort	IB1–IB2	59.1	2000–2018	122	122
Kim 2020	Korea	Retrospective cohort	Ia1—IIa1–Ib1–Ib2–IIa2	NR	2006–2016	26	22
Kong 2014	Korea	Retrospective cohort	IB–IIA	28/58	2006–2013	40	48
Lambaudie 2010	France	Prospective Cohort	IA1, IA2, IB1	NR	2007–2009	16	22
Laterza 2016	Italy	Retrospective cohort	IA1, IA2, IB1, IIA2	121.2/43.6	1997–2015	82	68
Lee 2002	Taiwan	Prospective cohort	IA2–IB	60	2002	30	30
Lee 2011	Korea	Retrospective cohort	IA2, IIA, IB1, IB2	78	1994–2001	24	48
Li 2021	China	Retrospective cohort	IA2–IB1–IIA1	33	2009–2016	546	661
Liang 2019	China	Retrospective cohort	IA1–IA2–IB1–IB2–IIA1–IIA2–IIB	NR	2004–2015	5491	12,956
Lim 2019	Singapore	Prospective cohort	IA1–IIA	29 (0–79)	2009–2014	51	85
Liu 2019	China	Retrospective cohort	IB1–IB2	NR	2001–2015	271	135
Malzoni 2009	Italy	Retrospective cohort	IA1 with LVSI–IB	53 (4–89)	1995–2007	65	62
Margina 2008	USA	Prospective cohort	IB, IC, IIA, IIB, IIIC	40	1993–2006	31	35
Mendivil 2016	USA	Retrospective cohort	IA2–IIB	39	2009–2013	39	49
Naik 2010	UK	Clinical trial	IB1	NR	2005–2007	7	6
Nam 2012	Korea	Retrospective cohort	IA2–IIA	92	1997–2008	263	263
Paik 2019	Korea	Retrospective cohort	IB–IIA	63.6	2000–2008	133	605
Park 2013	Korea	Retrospective cohort	IB2–IIA2	33	1997–2011	31	30
Park 2016	Korea	Retrospective cohort	IA2–IIA	58.8	1997–2013	186	107
Qin 2020	NR	Retrospective cohort	IA1, IA2, IB1	59	59	172	84
Rodriguez 2021	Colombia	Retrospective cohort	IA1–IIA2–IIB1	52.35	2006–2017	681	698
Sert 2011	Norway	Retrospective cohort	IA1–IB1 /IA1–IB1	63.2	2005–2009	7	26
Shanmugam 2020	India	Retrospective cohort	IA1, IA2, IB1, IB	33.5	2012–2018	82	89
Sharma 2006	England	Retrospective cohort	IA2–IIB	38.25	1999–2005	35	32
Soliman 2011	Brazil	Retrospective cohort	NR	NR	2007–2010	31	30
Steed 2004	Canada	Retrospective cohort	IA–IB	20	1996–2003	71	205
Suh 2015	Korea	Retrospective cohort	IA2–IIA	44	2003–2011	55	106
Taylor 2011	USA	Retrospective cohort	IA2–IB1 /IA2–IB1	NR	2003–2009	9	18
Topatas 2014	Turkey	Retrospective cohort	IA2–IB2	43	2007–2010	22	46
Wang 2019	China	Retrospective cohort	IA1, IA2, IB1, IB	41.3 (6–193.5)	2001–2015	217	179
Wright 2012	USA	Retrospective cohort	NR	NR	2006–2010	217	1610
Xiao 2015	China	Retrospective cohort	IA–IIB			106	48
Xiao 2016	China	Retrospective cohort	IA–IIA	48.6	2001–2014	42	16
Xu 2020	China	Clinical trial	IA1, IA2, IB1, IB	NR	NR	84	84
Yuan 2019	China	Retrospective cohort	IA2–IB2	64	2012–2014	99	99
Zhang 2017	China	Retrospective cohort	IA2–IIB	54	2008–2012	35	42
Zhao 2021	China	Retrospective cohort	IA2–IIA2	60	2013–2016	148	939

*NR* not reported, *mo* month.

**Table 2:** Baseline characteristics of participants.

Study ID	Age (years), (mean, SD) (median, range)		BMI, (mean, SD) (median, range)		Squamous n (%)		Adenocarcinoma n (%)		Positive lymph nodes n (%)	
	LH	OH	LH	OH	LH	OH	LH	OH	LH	OH
Abu-Rustum 2003	42.6 (30–69)	43.6 (20–85)	23.1 (18–30)	24.6 (18–40)	10 (53%)	132 (67%)	7	55	NR	NR
Anagnostopoulos 2017	44.6 (12.2)	41.2 (12.7)	25.8 (3.8)	26.4 (4.7)	25 (69%)	20 (55%)	11 (31%)	16 (45%)	NR	NR
Anchora 2019	46 (19–85)	46 (26–83)	29 (17–40)	26 (18–39)	139 (67.5)	141 (65.0	67 (32.5)	76 (35.0)	17 (8.3)	43 (19.8)
Bogani 2014	48.9 (± 13.5)	50.9 (± 14)	25.1 (± 5.2)	25.9 (± 6.1)	20 (31%)	22 (34%)	45 (69%)	43 (66%)	18 (28%)	17 (26%)
Bogani 2020	45 (25–82)	45 (25–68)	22.4 (15.8–39.8)	23.1 (15.7–33.3)	24 (68%)	59 (84%)	7 (20%)	8 (11%)	10 (28%)	28 (40%)
Campos 2013	36.19 ± 9.78	39.64 ± 6.23	NR	NR	12 (75%)	12 (86%)	4 (25%)	2 (14%)	NR	NR
Campos 2021	NR	NR	NR	NR	NR	NR	NR	NR	NR	NR
Chen 2014	51.2 (11.9)	51.9 (11.3)	23.2 (3.4)	24.9 (4.6)	26 (81.3)	33 (75)	1 (3.1)	1 (2.3)	3 (9.4)	9 (20.5)
Chen 2019	49.29 ± 9.31	51.69 ± 10.25	22.99 ± 3.29	22.98 ± 3.14	103 (79.84)	165 (84.18)	19 (14.73)	23 (11.73)	1 (0.78)	11 (5.61)
Chen 2020	47.0 ± 9.3	46.7 ± 9.6	NR	NR	784 (100%)	1393 (100%)	0	0	78 (9.9%)	106 (7.6%)
Chen 2021	49.00 ± 12.00	48.00 ± 14.00	NR	NR	66 (75.86)	140 (80.46)	18 (20.69)	27 (15.52)	4 (4.60)	8 (4.60)
Corrado 2018	45 (23–78)	50 (28–76)	23.5 (17–35)	24.8 (18–51)	110 (72.3%)	68 (67.3%)	37 (24.3%)	23 (22.8%)	148 (97.3%)	97 (96%)
Estape 2009	52.8 (4.8)	42 (12)	28.1 (4.8)	29.5 (6.4)	11 (64.7%)	10 (71.4%)	6 (35.3%)	2 (14.3%)	NR	NR
Ditto 2015	46 (29.79)	45.5 (15.78)	24.3 (2.9)	24.0 (4.3)	36 (60%)	35 (58%)	24 (40%)	25 (42%)	3 (5%)	6 (10%)
Frumovitz 2007	40.8 (28–63)	42.5 (27–68)	28.1 (18–40)	28.2 (17–46)	15	33	17	16	NR	NR
Ghezzi 2007	47 (24–78)	53 (28–75)	23 (17.4–35)	25 (19–43)	38 (76%)	33 (68.7%)	7 (14%)	13 (27.1%)	7 (14%)	9 (18.7%)
Gil-Moreno 2018	46.31 (11.04)	50.5 (13.6)	26 (18–38)	26.5 (18–40)	57 (63.3)	47 (61.8)	27 (30)	23 (30.2)	10 (11.11)	12 (15.79)
Gortchev 2012	42.5 ± 9.9	49.0 ± 11.0	NR	NR	41 (89.1%)	167 (95.4%)	5 (10.9%)	8 (4.6%)	5 (10.9%)	42 (24.0%)
Guangyi 2007	42 ± 9	44 ± 11	NR	NR	81	25	5	4	18	4
Guo 2018	44.19 (25–76)	40.52 (23–62)	22.81 (14.33–35.61)	23.19 (13.88–36.63)	340 (82.52)	110 (79.14)	72 (17.48)	29 (20.86)	53 (12.86)	20 (14.39)
He 2020	46.80 ± 9.460	46.69 ± 9.367	NR	NR	641	641	89	89	71	71
Kanao 2019	44.0 ± 10.2	49.0 ± 11.5	20.5 (19.1–23.3)	21.4 (19.7–23.7)	37 (46.3)	44 (53.0)	43 (53.7)	39 (47.0)	9 (11.2)	12 (14.5)
Kim 2018	40 sd is NR	45	NR	NR	NR	NR	NR	NR	NR	NR
Kim 2019	49.5 ± 11.2	49.0 ± 11.0			148 (66.7)	167 (75.2)	62 (27.9)	42 (18.9)	19 (8.6)	23 (10.4)
Kim 2020	48.77 (11.82)	53.82 (11.13)	23.80 (3.47)	24.43 (2.77)	21 (80.8)	15 (68.2)	5 (19.2)	6 (27.3)	NR	NR
Kong 2014	45.0 ± 10.6	48.0 ± 11.0	22.3 ± 2.9	23.4 ± 3.3	30 (75.0)	39 (81.3)	7 (17.5)	7 (14.6)	7 (17.5)	8 (16.7)
Lambaudie 2010	45 (32–57)	53 (31–72)	21.9 (14.3–39.4)	21.9 (17.2–34)	11 (68.7%)	17 (85.0%)	4 (25.0%)	3 (15.0%)	NR	NR
Laterza 2016	43 (24–77)	48 (26–85)	23.44 (16.9–39.76)	24.52 (19.3–43.3)	NR	NR	NR	NR	2 (2.4)	1 (1.6)
Lee 2002	46.2 ± 7.2	48.0 ± 6.8	NR	NR	27	25	3	5	14	5
Lee 2011	48.4 (39–68)	50.2 (34–67)	23.4 (18.2–32.4)	23.9 (15.8–34.6)	19 (79.2%)	38 (79.2%)	4 (16.7%)	8 (16.7%)	4 (16.7%)	10 (20.8%)
Li 2021	46.94 ± 9.367	47.03 ± 9.354	NR	NR	451 (85.6)	565 (58.48)	82 (15.02)	78 (11.8)	51 (9.34)	46 (6.96)
Liang 2019	NR	NR	NR	NR	4691 (85.4)	11,404 (88)	559 (10.2)	1006 (7.8)	NR	NR
Lim 2019	47 (28–70)	49 (30–70)	22.9 (12.9–33.7)	23.4 (14.7–33.9)	21 (41.2)	50 (58.8)	25 (49.0)	27 (31.8)	7 (13.7)	12 (14.1)
Liu 2019	42.9 ± 9.1	42.6 ± 7.9	23.1 ± 2.8	23.7 ± 3.0	217 (80.1)	119 (88)	42 (15.5)	12 (8.9)	42 (15.5)	15 (11.1)
Malzoni 2009	40.5 ± 7.7	42.7 ± 8.6	26 (19–35)	29 (19–35)	56 (86)	53 (85)	7 (10.5)	6 (10)	23.5 ± 5.1	25.2 ± 6.2
Margina 2008	54.9 (14.3)	50.9 (8.6)	26.8 (4.6)	27.3 (5.8)	12	14	6	7	NR	NR
Mendivil 2016	47.8 ± 12.02	51.3 ± 12.48	27.9 ± 5.71	29.2 ± 6.00	38 (69)	27 (77)	9 (18)	5 (12.8)	NR	NR
Naik 2010	38.5 (33.5–53.5)	37 (29.5–46)	24.8 ± 1.3	25.0 ± 1.8	6 (85)	5 (83)	1 (14)	1 (16)	NR	NR
Nam 2012	46.4	46.5	23.9	23.2	214 (81.4)	207 (78.7)	41 (15.6)	46 (17.5)	252 (95.8)	252 (95.8)
Paik 2019	45.2 ± 10.8	48.9 ± 11.2	NR	NR	91 (68.4)	453 (74.9)	42 (31.6)	152 (25.1)	0	0
Park 2013	48.5 (25–77)	48.1 (25–84)	23.1 (15.6–34.8)	23.7 (17.6–34.7	90 (78.3)	154 (81.9)	25 (21.7)	34 (18.1)	46 (40)	71 (37.8)
Park 2016	45.3 (27–71)	47.3 (28–73)	23.69 (17.1–34.9)	23.58 (17.1–35.9)	0 (0)	0 (0)	186 (100)	107 (100%)	29 (15.6)	16 (15.0)
Qin 2020	44.3 ± 8.2	42.8 ± 8.3	23.1 ± 2.8	23.2 ± 3.0	72 (85.7)	72 (85.7)	35 (20.3)	35 (20.3)	15 (8.7)	9 (10.7)
Rodriguez 2021	NR	NR	NR	NR	451 (66.2%)	462 (66.3%)	206 (30.3%)	208 (29.8%)	77 (11.3)	56 (8)
Sert 2011	45.0 ± 12.9	44.8 ± 11.8	22.5 ± 1.84	25 ± 3.0	5 (71.4)	19 (73)	2 (28.6)	6 (23)	NR	NR
Shanmugam 2020	52.5	50.3	NR	NR	74 (43.3%)	79 (46.2%)	7 (4.1%)	7 (4.1%)	7 (12.1%)	4 (6.8%)
Sharma 2006	43.4 (28–60)	42.8 (28–66)	NR	NR	18 (51.4)	16 (50)	9 (25.7)	11 (34.3)	NR	NR
Soliman 2011	44.2 (23.55–64.9)	48.1 (25.5–82.2)	29.5 (18.7–47.8)	26.2 (20.9–44.5)	16 (51.6)	13 (43.33)	12 (38.7)	16 (53.3)	3 (10)	9 (31)
Steed 2004	43 (30–69)	44 (24–86)	NR	NR	31 (44)	111 (54)	40 (56)	94 (46)	5 (7)	18 (9)
Suh 2015	49.1 ± 11.3	48.7 ± 11	23.1 ± 3.25	23.55 ± 3.7	NR	NR	NR	NR	5 (9)	39 (36.8)
Taylor 2011	41.4 (31–60)	41.1 (25–61)	26.3 (20.6–36.1)	26.9 (17–38.3)	5 (55.5)	11 (61.1)	4 (44.44)	7 (38.88)	NR	NR
Topatas 2014	46.5 (40–57)	50 (46–58)			18 (81.8)	29 (63.0)	1 (4.6)	5 (10.9)	2 (9.1)	3 (6.7)
Wang 2019	45.95 ± 7.331	44.76 ± 7.743	23.25 ± 2.629	23.56 ± 3.286	188 (86.6)	160 (89.4)	25 (11.5)	9 (5.0)	87 (40.1)	71 (39.7)
Wright 2012	NR	NR	NR	NR	NR	NR	NR	NR	NR	NR
Xiao 2015	43.7 ± 9.3	45.7 ± 11.3	23.8 ± 3.9	24.7 ± 3.8	96 (90.6%)	42 (87.5%)	6 (5.7%)	5 (10.4%)	9 (8.5%)	9 (18.8%)
Xiao 2016	47.1 ± 9.9	55.1 ± 7.6	23.0 ± 3.0	23.8 ± 2.6	42 (100)	16 (100)	0 (0)	0 (0)	NR	NR
Xu 2020	46.67 ± 9.49	44.9 ± 8.08	NR	NR	65	64	14	16	NR	NR
Yuan 2019	43.58 ± 8.86	44.56 ± 7.60	44.56 ± 7.60	24.56 ± 1.50	82 (82.8%)	82 (82.8%)	14 (14.1%)	13 (13.1%)	11 (11.1%	10 (10.1%)
Zhang 2017	45 (29–64)	46.6 (27–75)	22.68 ± 3.15	24.07 ± 3.3	32 (91.4)	40 (95.2)	3 (5.6)	2 (4.8)	NR	NR
Zhao 2021	47.02 ± 8.70	49.53 ± 9.32	NR	NR	NR	NR	NR	NR	NR	NR

*NR* not reported, *n* number, *SD* standard deviation.

### Results of risk of bias assessment

The result of the quality assessment yielded an overall moderate risk of bias according to Cochrane`s tool and the mean score for observational studies was 10.57 out of 14 according to NHLB. All clinical trials^44,55,56,83^ reported proper randomization therefore they were categorized as low risk of bias. All clinical trials were at low risk of bias regarding attrition bias and selective reporting. A detailed illustration of the quality assessment of the included studies is illustrated in Supplementary Table S2.

### Analysis of outcomes

#### Operative time (minutes)

**Figure 2:**
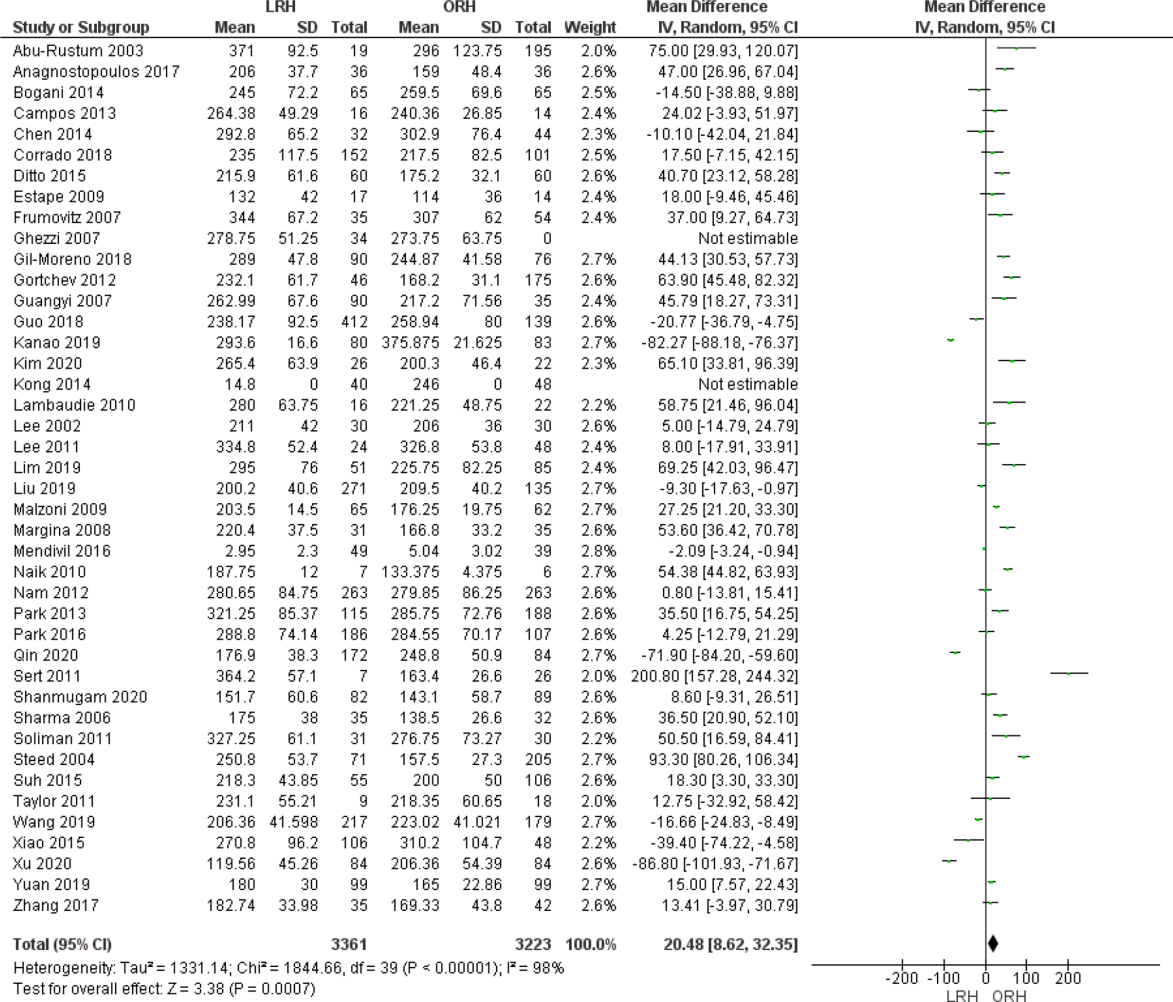
Forest plot of operative time.

Operating time was reported by 42 studies. We found that ORH operating time was significantly lower than LRH operative time (MD = 20.48 [8.62, 32.35], (*P* = 0.007). Pooled data were heterogeneous (*P* < 0.001); I^2^ = 98% which could not be solved by the leave-one-out method or subgroup analysis (Fig. 2).

#### Estimated blood loss (ml)

**Figure 3:**
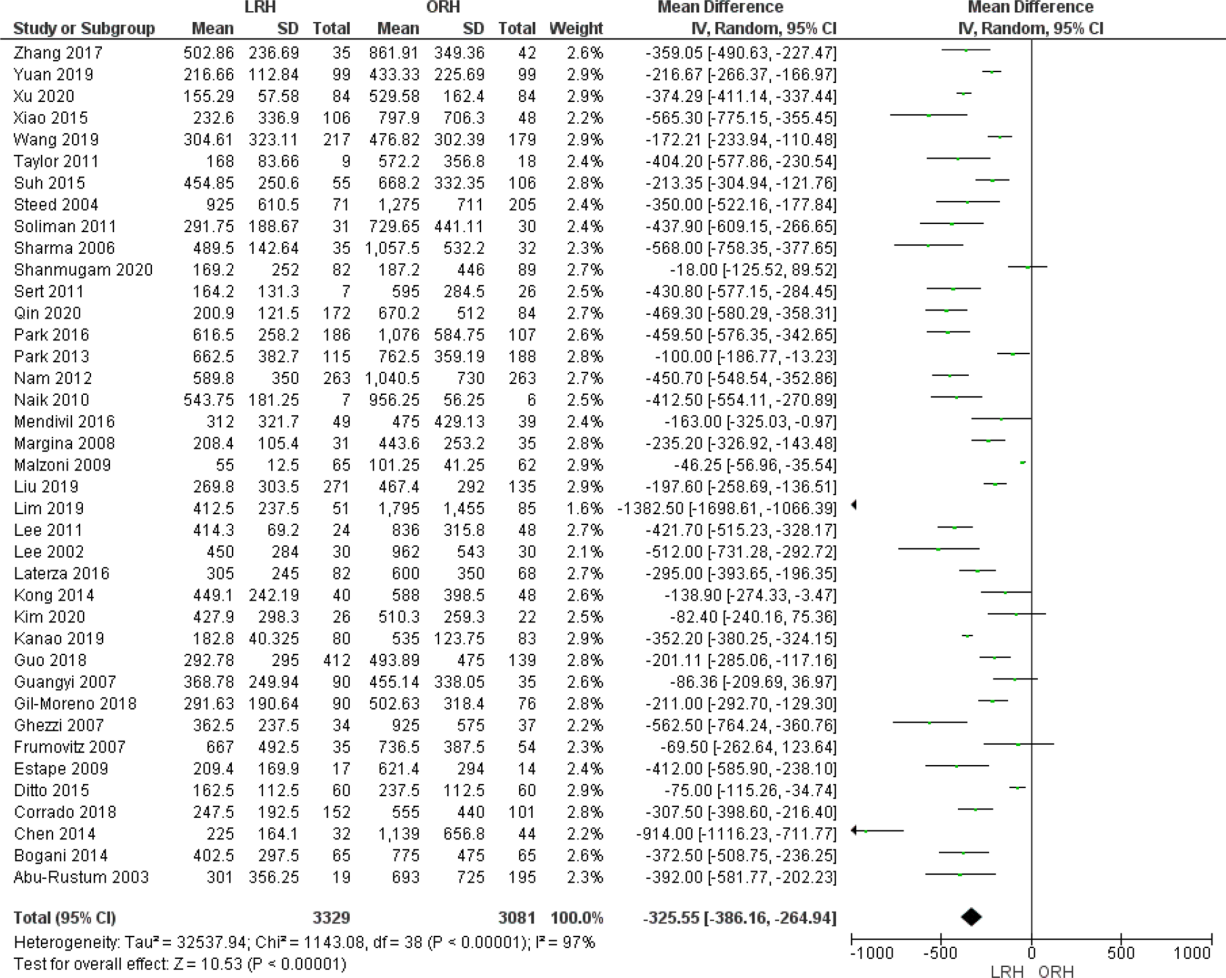
Forest plot of estimated blood loss (EBL).

We analyzed 6410 patients from 39 studies that reported the estimated blood loss. The overall mean difference showed that blood loss was significantly lower in LRH group than ORH group (MD = − 325.55 [− 386.16, − 264.94] (*P* < 0.001)), Pooled analysis was heterogeneous (*P* < 0.001); I^2^ = 97% (Fig. 3).

#### Intraoperative complication

**Figure 4:**
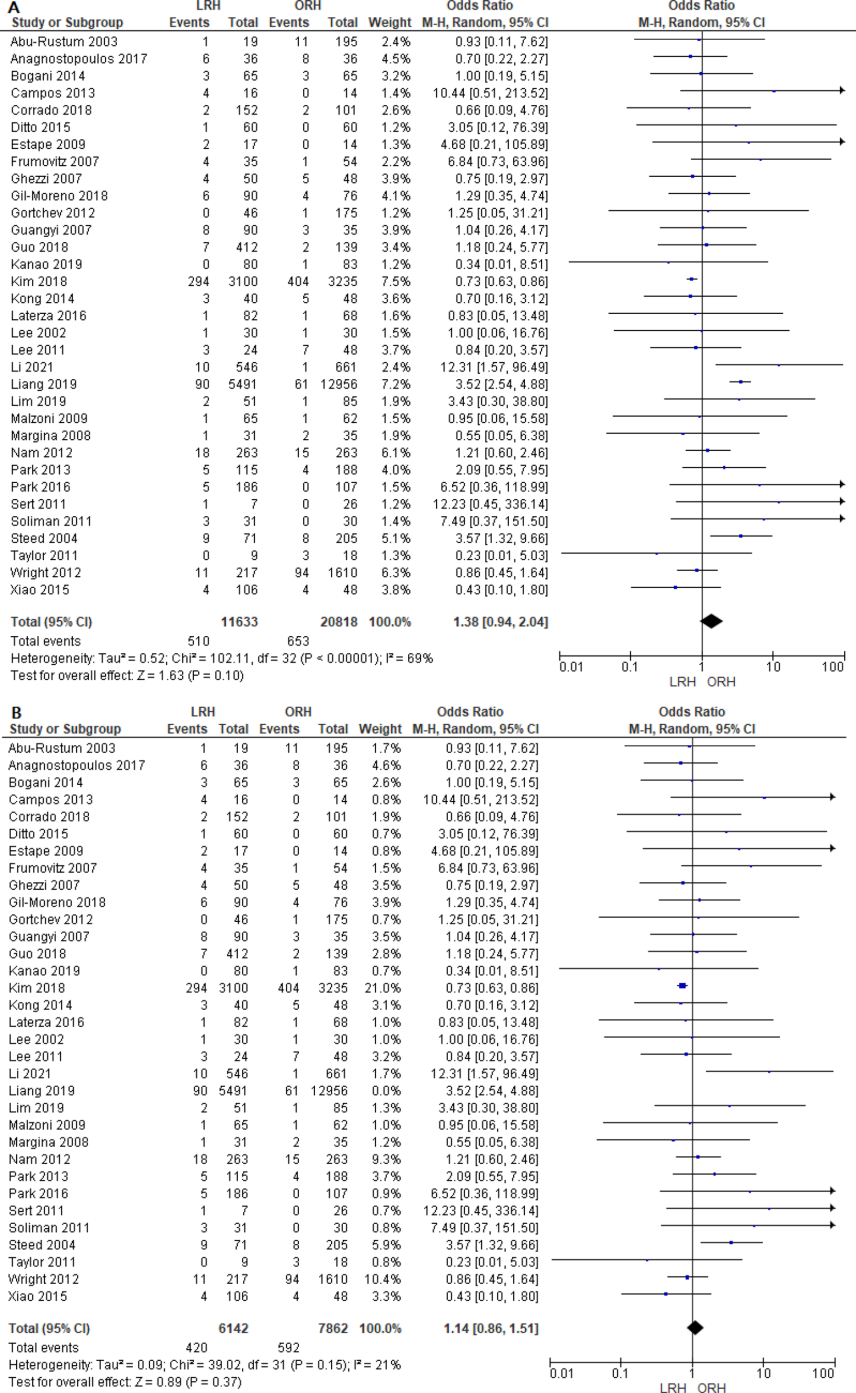
Forest plot of the rate of intraoperative complications before (**A**) and after (**B**) solving for heterogeneity by excluding the Liang et al. study.

Thirty two studies reported the rate of intraoperative complications. We found no significant difference between both groups (OR = 1.38 [0.94, 2.04], (*P* = 0.10)). Data was heterogeneous (*P* < 0.001); I^2^ = 69% as shown in Fig. 4A. We solved the heterogeneity by excluding Liang et al.^87^ (*P* = 0.15); I^2^ = 21%, the overall odds ratio after solving heterogeneity did not show any significant difference between both groups (OR = 1.14 [0.86, 1.51], (*P* = 0.37)) (Fig. 4B).

#### Postoperative complication

**Figure 5:**
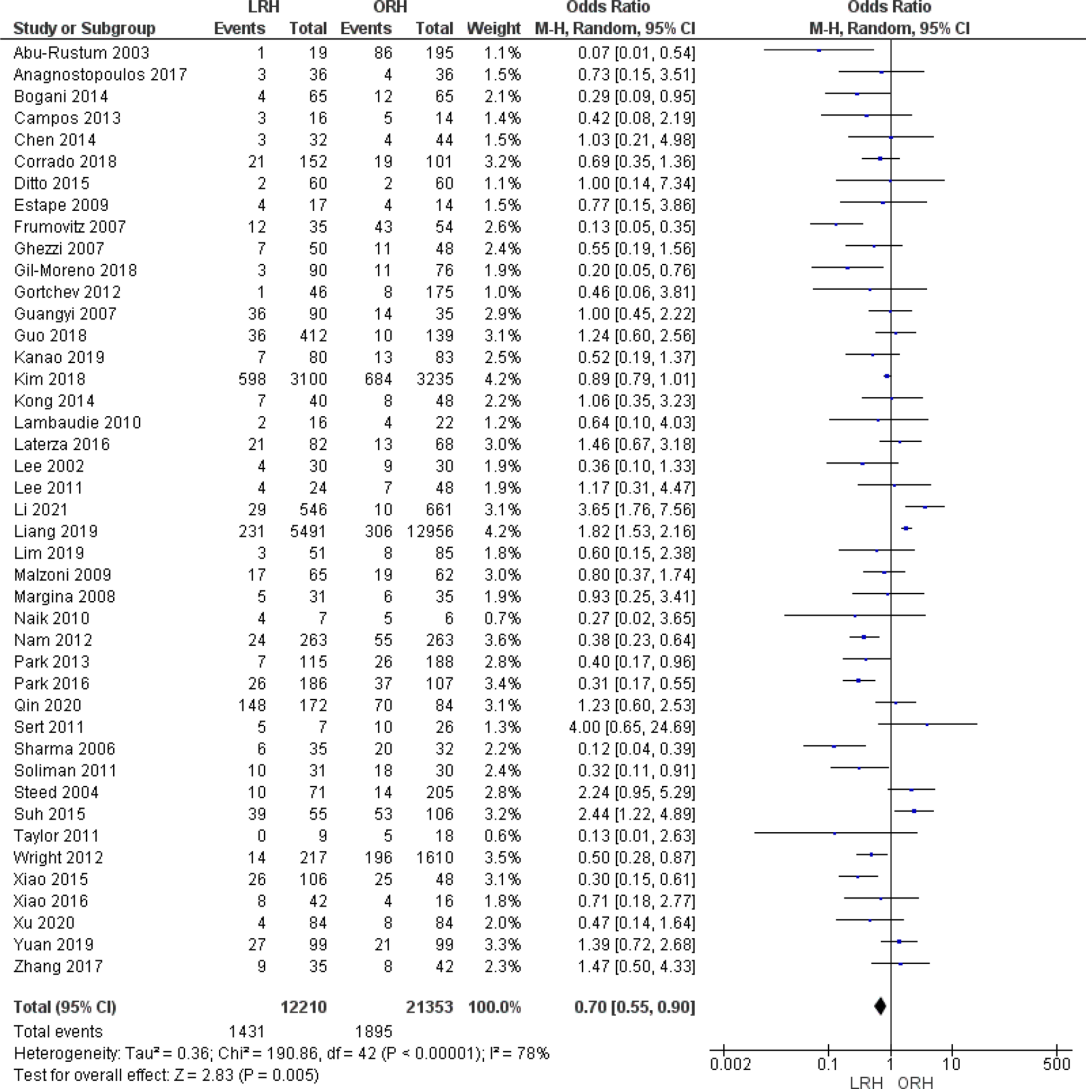
Forest plot of the rate of postoperative complications.

A total of 33,563 patients were analyzed from 43 studies that reported postoperative complications. The overall odds ratio showed that the LRH group had a postoperative complications rate significantly lower than that of ORH (OR = 0.70 [0.55, 0.90], (*P* = 0.005)). Pooled data was heterogeneous (*P* < 0.001); I^2^ = 78%. We could not solve heterogeneity by the leave-one-out method or subgroup analysis (Fig. 5).

#### Length of hospital stay (days)

**Figure 6:**
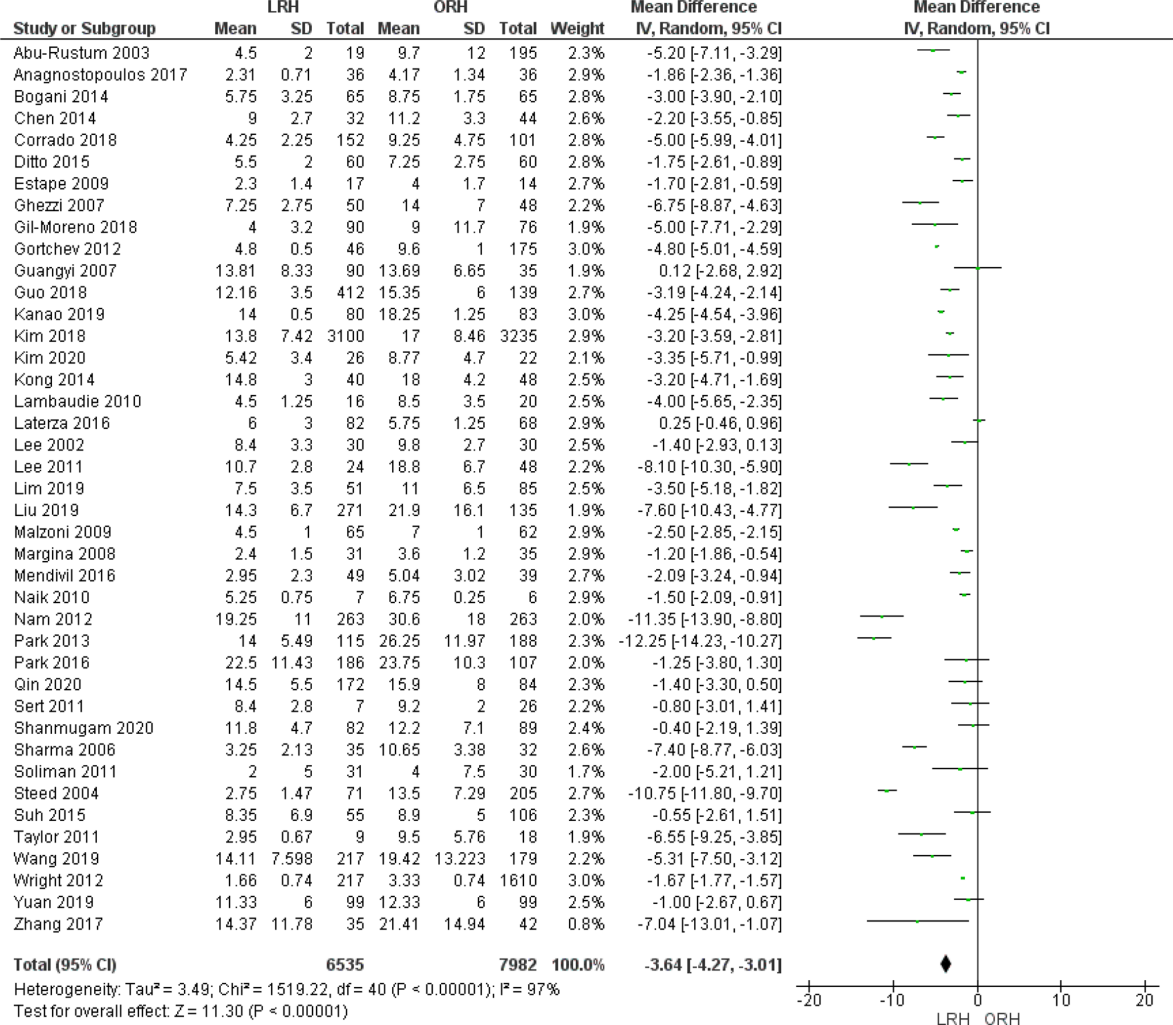
Forest plot of the average length of hospital stay.

Forty-one studies reported length of hospital stay. We found that patients in the LRH group stayed at hospital fewer days than patients in the ORH group (MD = − 3.64 [− 4.27, − 3.01], (*P* < 0.001)). We found heterogeneity which could not be solved by the leave-one-out method or subgroup analysis (Fig. 6).

#### Resected lymph nodes

**Figure 7:**
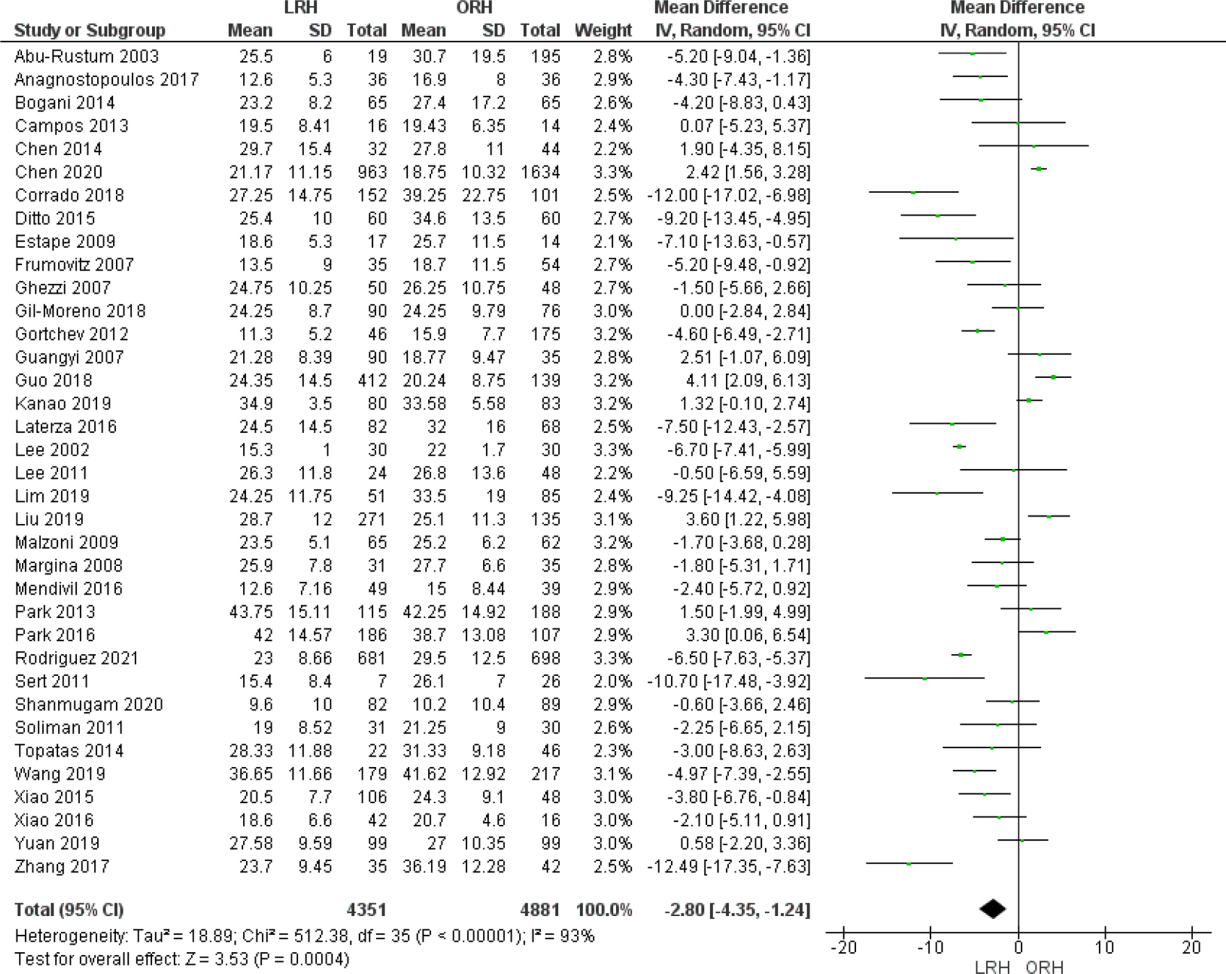
Forest plot of the number of resected lymph nodes.

Thirty six studies reported the number of resected lymph nodes as an outcome. Pooled analysis showed that the LRH group was associated with fewer resected lymph nodes than the ORH group (MD = − 2.80 [− 4.35, − 1.24], (*P* = 0.004)). We found heterogeneity among studies (*P* < 0.001); I^2^ = 93% (Fig. 7).

#### Five-year Overall Survival

**Figure 8:**
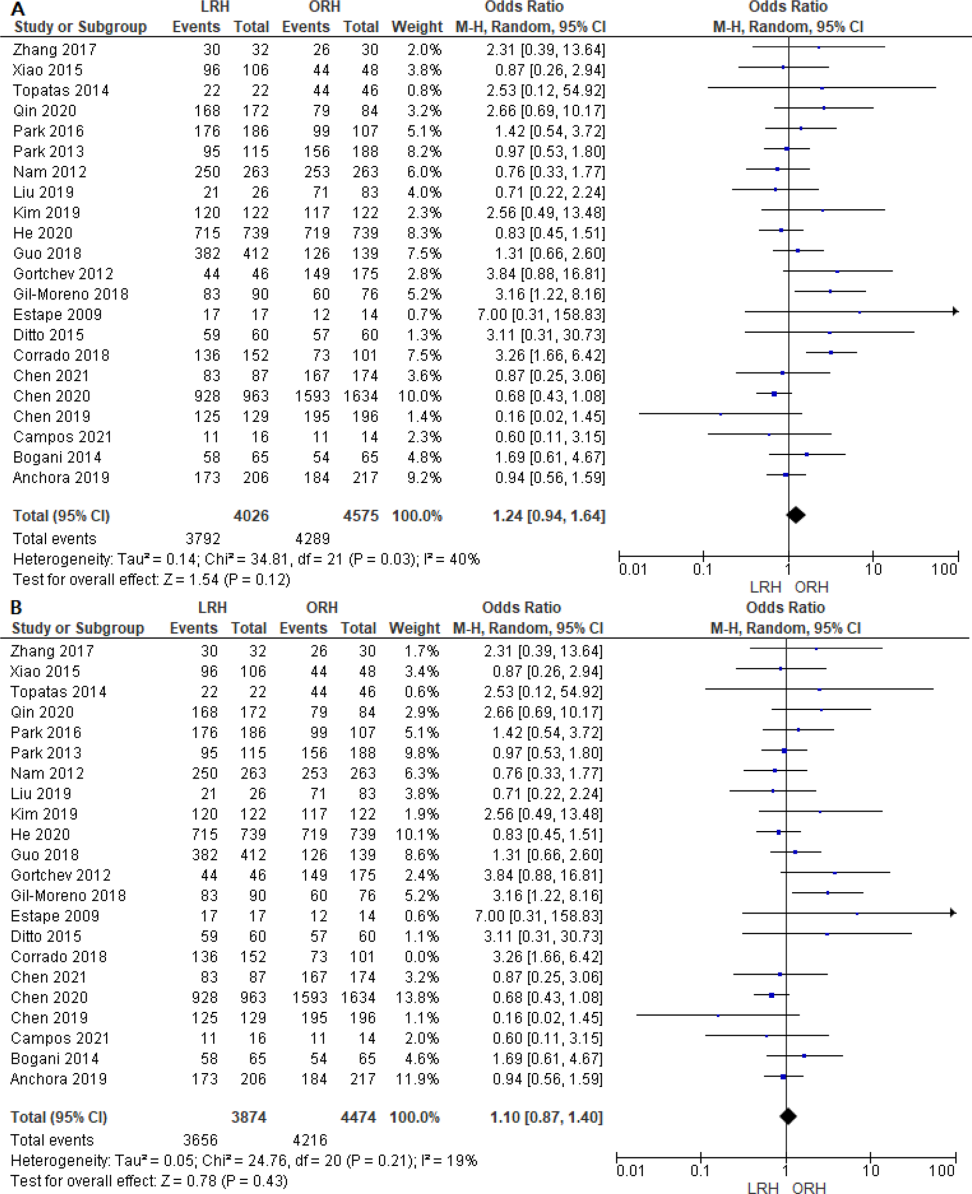
Forest plot of five-year overall survival before (**A**) and after (**B**) solving for heterogeneity by excluding the Corrado et al. study.

A total of 8610 patients were analyzed from 22 studies. The combined analysis did not show any significant difference between both groups (OR = 1.24 [0.94, 1.64], (*P* = 0.12)). A little heterogeneity was found among studies (*P* = 0.03); I^2^ = 40% (Fig. 8A). We solved the heterogeneity by excluding Corrado et al.^59^ (*P* = 0.21); I^2^ = 19%. The overall analysis after solving heterogeneity also showed no significant difference between both groups (OR = 1.10 [0.87, 1.40], (*P* = 0.43)) (Fig. 8B).

#### Disease free survival

**Figure 9:**
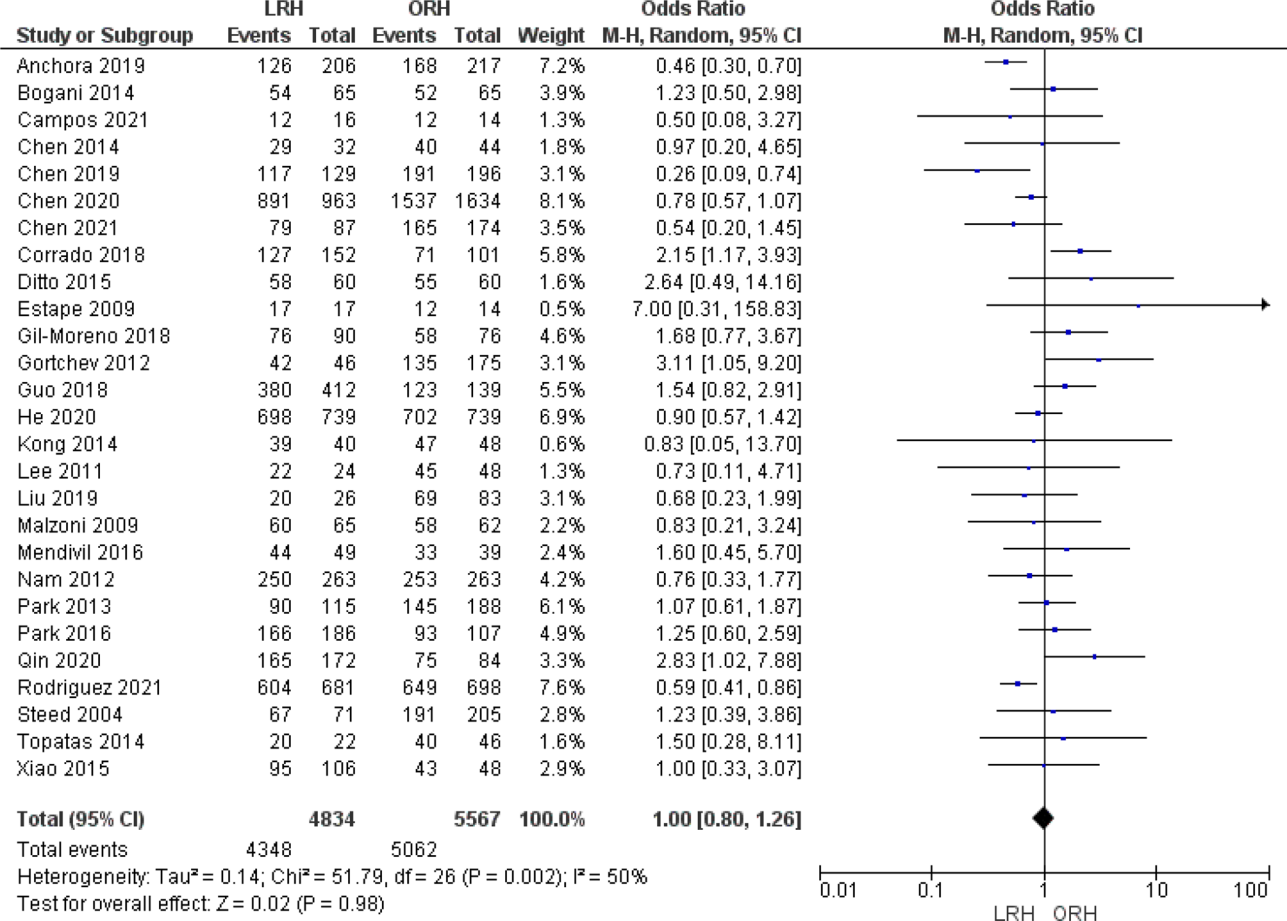
Forest plot of the rate of disease free survival.

Twenty seven studies reported DFS. Pooled analysis did not show any difference between both groups (OR = 1.00 [0.80, 1.26], (*P* = 0.98)). Data was heterogeneous (*P* = 0.002); I^2^ = 50% (Fig. 9).

#### Postoperative or Intraoperative Mortality (Defined as within 90 days postop)

**Figure 10:**
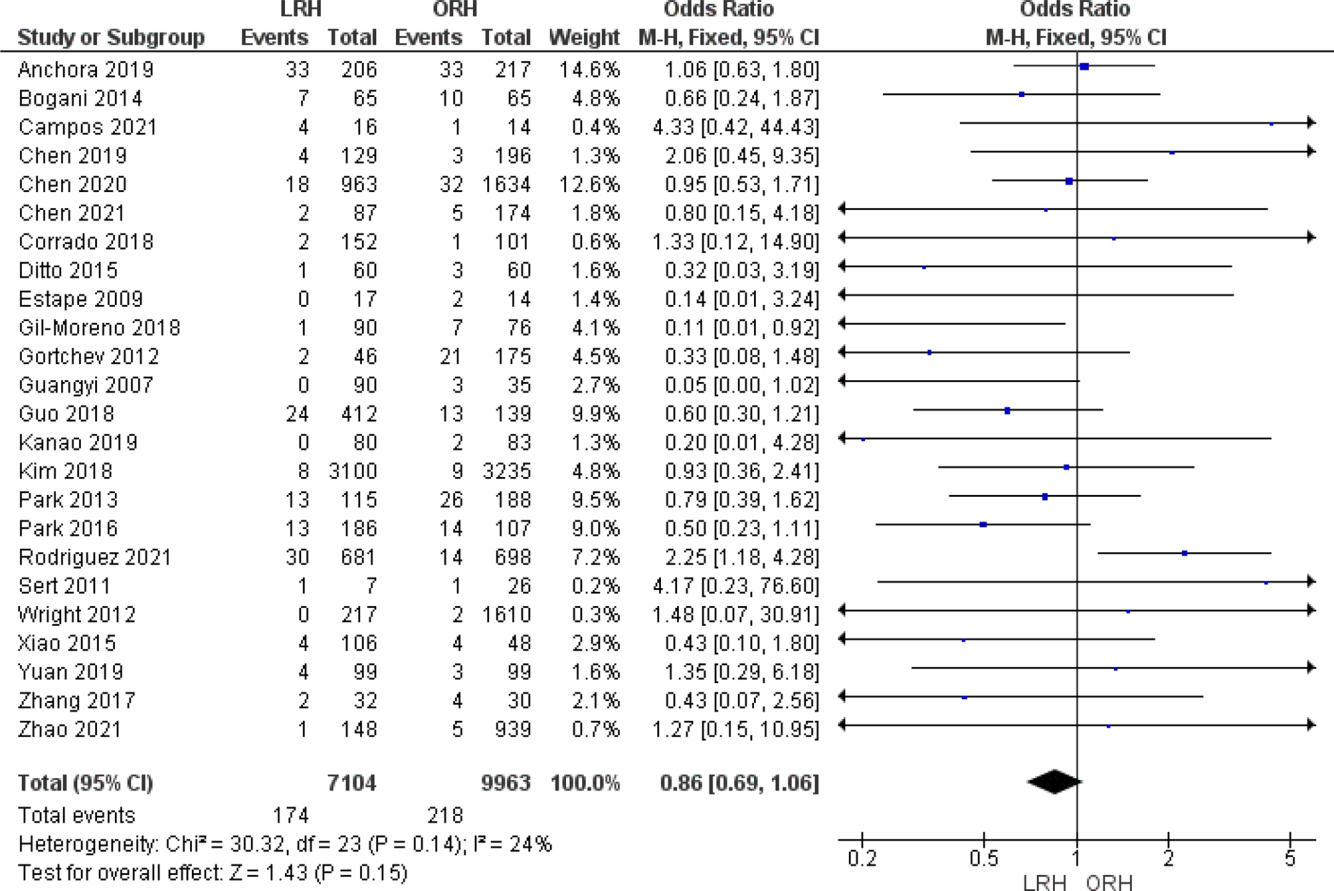
Forest plot of the postoperative or intraoperative mortality rate.

Twenty four studies reported mortality as an outcome. The overall odds ratio showed no significant difference between either group (OR = 0.86 [0.69, 1.06], (*P* = 0.15). Pooled analysis was homogeneous (*P* = 0.14); I^2^ = 24% (Fig. 10).

#### Recurrence

**Figure 11:**
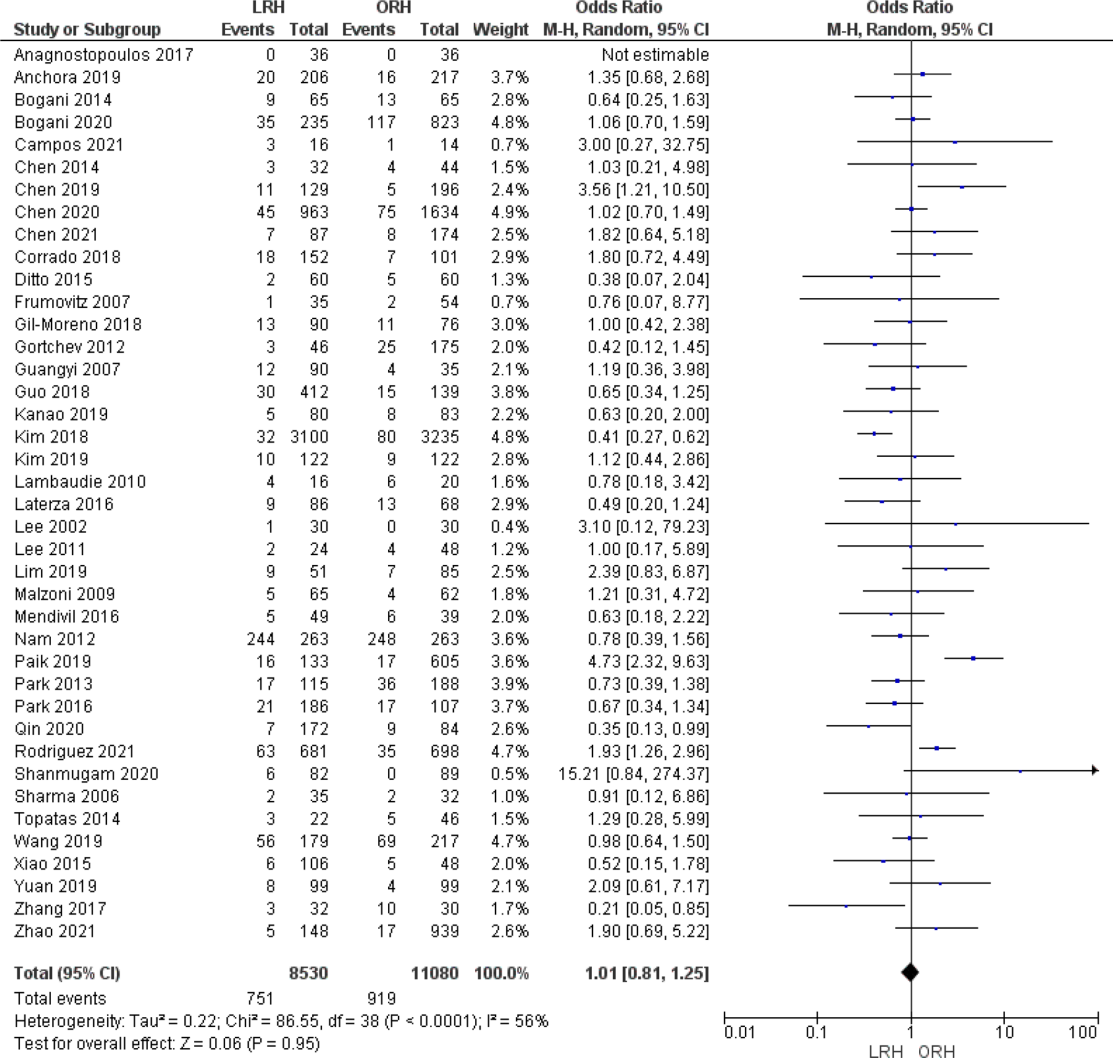
Forest plot of recurrence of disease.

A total of 19,610 patients were analyzed from 40 studies. We found no significant difference between the two groups (OR = 1.01 [0.81, 1.25], (*P* = 0.95). Data was heterogeneous (*P* < 0.001); I^2^ = 56% (Fig. 11).

#### Blood transfusion rate

**Figure 12:**
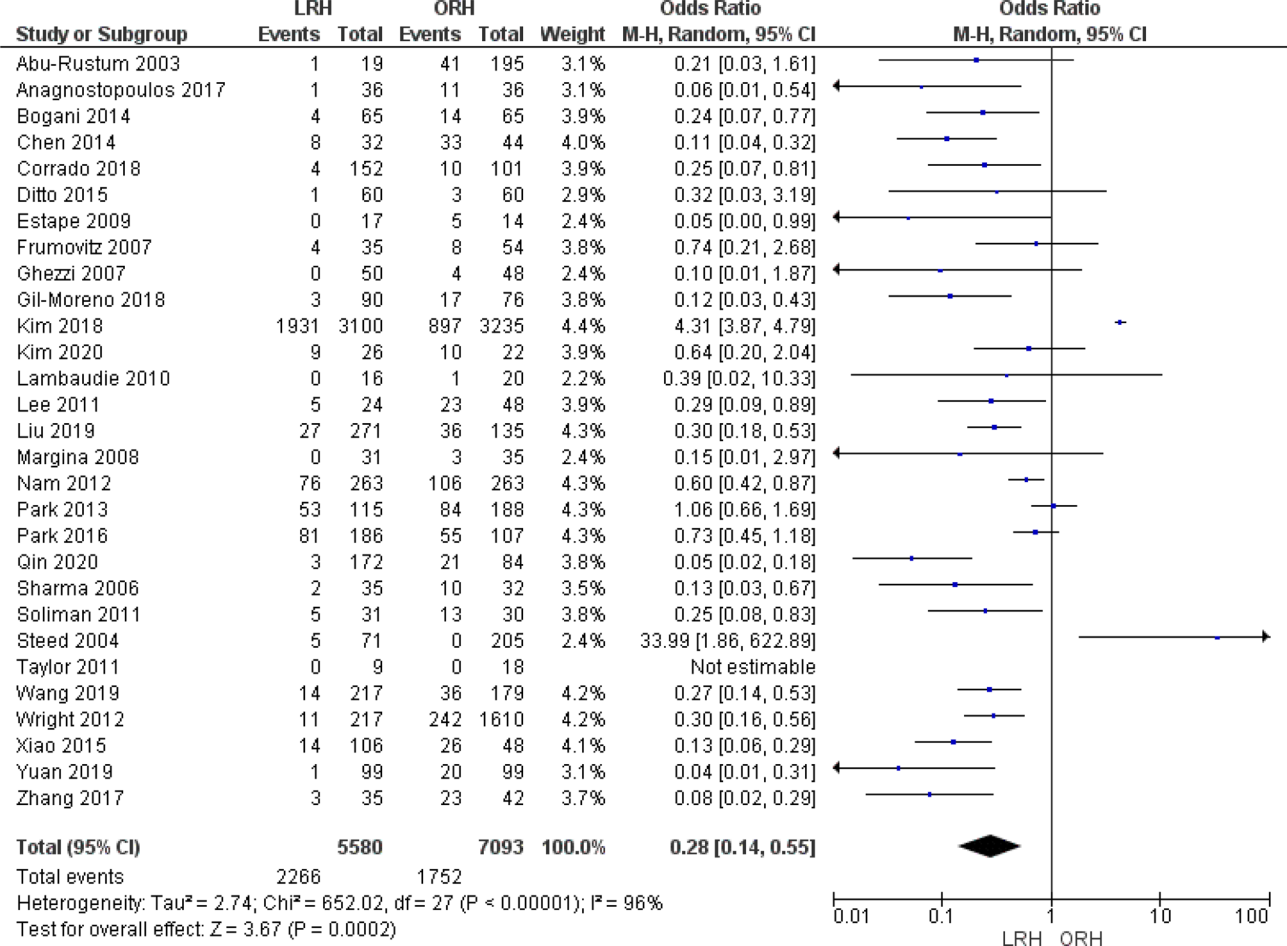
Forest plot of the rate of blood transfusion.

The combined analysis of 12,673 patients from 29 studies favored the LRH group significantly (OR = 0.28 [0.14, 0.55], (*P* = 0.002). Pooled analysis was heterogeneous (*P* < 0.001); I^2^ = 96% (Fig. 12).

A funnel plot and Egger test of all outcomes can be found in Supplementary File S1. A separate sensitivity analysis involving only the studies with a low risk of bias can also be found in Supplementary File S2. The sensitivity analysis showed different findings only in the outcome of postoperative complications, where the result changed from statistically significant to insignificant. There were no changes in any other outcomes within the sensitivity analysis.

## Discussion

This is the largest scale meta-analysis to date that has compared LRH to ORH through an evaluation of the available evidence, and the first, to our knowledge, to attempt to do so while specifically excluding robotic assisted techniques. In our study, we found that LRH was associated with a significantly lower incidence of estimated blood loss, postoperative complications. In addition, predictably, the LRH group had a short period of postoperative hospital stay. The number of the resected in the LRH group lymph node was significantly fewer than that of the ORH group. The combined analysis did not show any significant difference between both groups regarding 5-year overall survival, disease-free survival, and postoperative or intraoperative mortality. The duration of the surgical procedure was significantly longer in the LRH compared to ORH. This stands in contrast to previous studies that showed decreased survival as a result of minimally invasive radical hysterectomy techniques^20^.

Although isolated studies have disagreed with these results, the majority of previous systematic reviews and meta-analyses have not. In 2015, Wang et al.^88^ performed a meta-analysis of 12 cohort studies. They demonstrated similar results to our study. They conclude that LRH was better than ORH regarding the short-term outcomes such as faster functional recovery, estimated blood loss, and postoperative complications. The survival outcomes were also similar in both groups.

Smith et al.^89^ performed another meta-analysis to compare the minimally invasive hysterectomy with abdominal radical hysterectomy. They found that in more than 22,000 women with early-stage cervical cancer the progression-free survival was significantly worse for women who underwent the minimally invasive radical hysterectomy. This finding is strengthened with longer follow-up. The pooled relative risk for postoperative or intraoperative mortality was lower in the minimally invasive group.

In 2020, Kampers et al.^90^ performed the first meta-analysis stratifying the patients subjected to different operation techniques according to their risk factors. They compared the survival rates of open hysterectomy and laparoscopic hysterectomy in different risk groups. The study demonstrated that protective techniques in laparoscopy result in improved survival.

Kong et al.^84^ conducted a retrospective analysis of 88 patients with a cervical cancer diameter of 3 cm or greater. They found that LRH can be a feasible alternative surgical procedure for the management of FIGO stage IB and IIA cervical cancer. However, the institution at which the study was performed was introduced with the laparoscopic approach much later than the laparotomy approach. This in turn made the follow-up period in the LRH group relatively short to make any conclusive remarks on survival benefits.

Liang et al.^87^ reviewed the records of 18,447 patients undergoing radical hysterectomy for cervical cancer. They demonstrated that laparoscopic hysterectomy was associated with a higher risk of major complications than conventional laparotomy. Prior to our review, this study probably represented the largest scale evidence that assessed surgical complications after radical hysterectomy. Other studies to attempt this were single-center studies with small sample sizes^15,60,74^. In reconciling why the findings of Liang et al. did not also appear as statistically significant in our analysis, we hypothesize that this had to do with limitations on Liang’s study design. This study relied on inpatient medical records or readmission records to obtain information on complications without regular follow-up of the patients. Therefore, without any first-hand knowledge of the circumstances of this study we would hypothesize that perhaps this their conclusion may not have been as statistically significant if direct outreach to patients had been attempted, consistent with other cohort analyses.

In 2021, Campos et al.^55^ performed a single-center randomized controlled trial on 30 patients with cervical cancer and lymphovascular invasion. They demonstrated a non-significant trend of worse outcomes for LRH. The overall survival time and disease-free survival time were longer in the LRH group. However, the main limitation facing the study was the small sample size.

In 2018, the LACC study by Ramirez et al.^20^ found that the minimally invasive radical hysterectomy had lower rates of overall survival and disease-free survival than abdominal radical hysterectomy. It was largely based on this study that the National Comprehensive Cancer Network (NCCN) recommended careful counseling of the patient about short-term versus long-term outcomes and oncologic risks of the different surgical approaches.

## Limitations

Although the main limitation facing us in this study is the heterogeneity in some outcomes, we managed to understand most of the attributing factors. We believe the use of studies with different designs and disparity in follow-up periods was responsible for the heterogeneity, which is understandable. Another possible limitation of our study includes selection bias, which is difficult to account for. With observational studies, there is always the possibility that surgeons have intentionally selected out cases for laparoscopic radical approaches that appear easier or have different characteristics than those chosen to be performed open. This could affect data. Our analysis represents the most recent and wide-scale evidence that compares LRH to ORH among women with cervical cancer. In light of this controversy it would be desirable to have more well designed prospective randomized trials in order to strengthen the evidence and dissolve any remaining controversy.

## Conclusion

We conclude that LRH is associated with a significantly lower incidence of estimated blood loss, postoperative complications. In addition, the LRH had a short period of postoperative hospital stay. In contrast to previous studies that mixed robotic assisted cases we found no difference regarding 5-year overall survival and recurrence excluding robotic assisted cases.

## Supplementary Information

Supplementary Table S1.

Supplementary Table S2.

Supplementary Information 3.

Supplementary Information 4.

Supplementary Legends.

## Data Availability

The datasets used and/or analyzed during the current study available from the corresponding author on reasonable request.
