# Supplementary material for: Meta-analysis of laparoscopic radical hysterectomy, excluding robotic assisted versus open radical hysterectomy for early stage cervical cancer
Source: Sci Rep. 2023 Jan 6;13:273. doi: 10.1038/s41598-023-27430-9 (PMC9822966; doi:10.1038/s41598-023-27430-9)
Supplement: Supplementary file 1 — Supplementary Table S1. [file 41598_2023_27430_MOESM1_ESM.docx]

**Supplementary Table S1:** GRADE quality assessments for included studies

| Outcome name | Number of included studies | Design of included studies | Mean difference, 95% CI | Heterogeneity | Number of patients in Laparoscopic | Number of patients in Open | Risk of bias | Inconsistency | Indirectness | Imprecision | Other considerations | Quality |
| --- | --- | --- | --- | --- | --- | --- | --- | --- | --- | --- | --- | --- |
| Operative time | 42 | Observational | 20.48 [8.62, 32.35] | (P < 0.001); I² = 98% | 3361 | 3223 | Not serious | Serious | Not serious | Not serious | Not serious | Low |
|  |  |  |  |  |  |  |  |  |  |  |  | ⊕⊕◯◯ |
| Estimated blood loss | 39 | Observational | 325.55 [-386.16, -264.94] | (P < 0.001); I² = 97% | 3329 | 3081 | Not serious | Serious | Not serious | Not serious | Serious | Moderate |
|  |  |  |  |  |  |  |  |  |  |  |  | ⊕⊕⊕◯ |
| Intraoperative complication | 32 | Observational | 1.14 [0.86, 1.51] | (P < 0.001); I² = 69% | 6142 | 7862 | Not serious | Serious | Not serious | Not serious | Not serious | Low |
|  |  |  |  |  |  |  |  |  |  |  |  | ⊕⊕◯◯ |
| Postoperative complication | 43 | Observational | 0.70 [0.55, 0.90] | (P < 0.001); I² = 78% | 12210 | 21353 | Not serious | Serious | Not serious | Not serious | Serious | Moderate |
|  |  |  |  |  |  |  |  |  |  |  |  | ⊕⊕⊕◯ |
| Length of hospital stay | 41 | Observational | 3.64 [-4.27, -3.01] | (P < 0.001)) | 6535 | 7982 | Not serious | Serious | Not serious | Not serious | Serious | Moderate |
|  |  |  |  |  |  |  |  |  |  |  |  | ⊕⊕⊕◯ |
| Resected lymph nodes | 36 | Observational | 2.80 [-4.35, -1.24] | P = 0.004 | 4351 | 4881 | Not serious | Serious | Not serious | Not serious | Serious | Moderate |
|  |  |  |  |  |  |  |  |  |  |  |  | ⊕⊕⊕◯ |
| 5 year Overall Survival | 21 | Observational | 1.10 [0.87, 1.40] | (P = 0.43) | 3874 | 4474 | Not serious | Not serious | Not serious | Not serious | Not serious | Low |
|  |  |  |  |  |  |  |  |  |  |  |  | ⊕⊕◯◯ |
| Disease free survival | 27 | Observational | 1.00 [0.80, 1.26] | (P = 0.002); I² = 50% | 4834 | 5567 | Not serious | Serious | Not serious | Not serious | Not serious | Low |
|  |  |  |  |  |  |  |  |  |  |  |  | ⊕⊕◯◯ |
| Mortality | 24 | Observational | 0.86 [0.69, 1.06] | (P = 0.14); I² = 24% | 7104 | 9963 | Not serious | Serious | Not serious | Not serious | Not serious | Low |
|  |  |  |  |  |  |  |  |  |  |  |  | ⊕⊕◯◯ |
| Recurrences | 40 | Observational | 1.01 [0.81, 1.25] | (P < 0.001); I² = 56% | 8530 | 11080 | Not serious | Serious | Not serious | Not serious | Not serious | Low |
|  |  |  |  |  |  |  |  |  |  |  |  | ⊕⊕◯◯ |
| Blood transfusion rate | 29 | Observational | 0.28 [0.14, 0.55] | (P < 0.001); I² = 96% | 5580 | 7093 | Not serious | Serious | Not serious | Not serious | Serious | Moderate |
|  |  |  |  |  |  |  |  |  |  |  |  | ⊕⊕⊕◯ |
