# Supplementary material for: Meta-analysis of laparoscopic radical hysterectomy, excluding robotic assisted versus open radical hysterectomy for early stage cervical cancer
Source: Sci Rep. 2023 Jan 6;13:273. doi: 10.1038/s41598-023-27430-9 (PMC9822966; doi:10.1038/s41598-023-27430-9)
Supplement: Supplementary file 2 — Supplementary Table S2. [file 41598_2023_27430_MOESM2_ESM.docx]

**Supplementary Table S2: Quality assessment for included retrospective studies**

|  | Abu-Rustum 2003 | Anagnostopoulos 2017 | Anchora 2019 | Bogani 2014 | Bogani 2020 | Chen 2014 | Chen 2019 | Chen 2020 | Chen 2021 | Corrado 2018 | Estape 2009 | Ditto 2015 | Zhao 2021 |
| --- | --- | --- | --- | --- | --- | --- | --- | --- | --- | --- | --- | --- | --- |
| 1.Was the research question or objective in this paper clearly stated? | 1 | 1 | 1 | 1 | 1 | 1 | 1 | 1 |  | 1 | 1 | 1 | 1 |
| 2.Was the study population clearly specified and defined? | 1 | 1 | 1 | 1 | 1 | 1 | 1 | 1 | 1 | 1 | 1 | 1 | 1 |
| 3.Was the participation rate of eligible persons at least 50%? | 1 | 1 | 1 | 1 | 0 | 1 | 1 | 1 | 1 | 1 | * | 1 | 1 |
| 4.Were all the subjects selected or recruited from the same or similar populations (including the same time period)? Were inclusion and exclusion criteria for being in the study prespecified and applied uniformly to all participants? | 0 | 1 | 1 | 1 | 1 | 1 | 1 | 1 | 1 | 0 | 0 | 1 | 1 |
| 5. Was a sample size justification, power description, or variance and effect estimates | 0 | 0 | 0 | 0 | 0 | 0 | 0 | 0 | 0 | 0 | 0 | 0 | 0 |
| 6.For the analyses in this paper, were the exposure (s) of interest measured prior to the outcome(s) being measured? | 1 | 1 | 1 | 1 | 1 | 1 | 1 | 1 | 1 | 1 | 1 | 1 | 1 |
| 7. Was the timeframe sufficient so that one could reasonably expect to see an association between exposure and outcome if it existed? | 1 | 1 | 1 | 1 | 1 | 1 | 1 | 1 | 1 | 1 | 0 | 1 | 1 |
| 8. For exposures that can vary in amount or level, did the study examine different levels of the exposure as related to the outcome (e.g., categories of exposure, or exposure measured as continuous variable)? | 1 | 1 | 1 | 1 | 1 | 1 | 1 | 1 | 1 | 1 | 1 | 1 | 1 |
| 9. Were the exposure measures (independent variables) clearly defined, valid, reliable, and implemented consistently across all study participants? | 1 | 1 | 1 | 1 | 1 | 1 | 1 | 1 | 1 | 1 | 1 | 1 | 1 |
| 10. Was the exposure(s) assessed more than once over time? | 0 | 0 | 1 | 0 | 0 | 0 | 0 | 0 | 0 | 0 | 0 | 0 | 0 |
| 11. Were the outcome measures (dependent variables) learly defined, valid, reliable, and implemented consistently across all study participants? | 1 | 1 | 1 | 1 | 1 | 1 | 1 | 1 | 1 | 1 | 1 | 1 | 1 |
| 12. Were the outcome assessors blinded to the exposure status of participants? | * | * | * | * | * | * | * | * | * | * | * | * | * |
| 13. Was loss to follow-up after baseline 20% or less? | 1 | 1 | 1 | 1 | 1 | 1 | 1 | 1 | 1 | 1 | 1 | 1 | 1 |
| 14. Were key potential confounding variables measured and adjusted statistically for their impact on the relationship between exposure(s) and outcome(s)? | 1 | 0 | 1 |  |  |  |  | 1 |  | 1 | 0 |  |  |
| Total score (out of 14) | 10/14 | 10/14 | 12/14 | 11/14 | 10/14 | 11/14 | 11/14 | 11/14 | 11/14 | 10/14 | 7/14 | 11/14 | 11/14 |

|  | Frumovitz 2007 | Ghezzi 2007 | Gil-Moreno 2018 | Gortchev 2012 | Guangyi 2007 | Guo 2018 | He 2020 | Kanao 2019 | Kim 2018 | Kim 2019 | Kim 2020 | Kong 2014 | Lambaudie 2010 | Laterza 2016 | Zhang 2017 |
| --- | --- | --- | --- | --- | --- | --- | --- | --- | --- | --- | --- | --- | --- | --- | --- |
| 1.Was the research question or objective in this paper clearly stated? | 1 | 1 | 1 | 1 | 1 | 1 | 1 | 1 | 1 | 1 | 1 | 1 | 1 | 1 | 1 |
| 2.Was the study population clearly specified and defined? | 1 | 1 | 1 | 0 | 1 | 1 | 1 | 1 | 1 | 1 | 1 | 1 | 1 | 1 | 0 |
| 3.Was the participation rate of eligible persons at least 50%? | 1 | 1 | 1 | 1 | 1 | 1 | 1 | 1 | 1 | 1 | 1 | 1 | 1 | 1 | 1 |
| 4.Were all the subjects selected or recruited from the same or similar populations (including the same time period)? Were inclusion and exclusion criteria for being in the study prespecified and applied uniformly to all participants? | 1 | 1 | 0 | 1 | 1 | 1 | 1 | 1 | 1 | 1 | 1 | 1 | 0 | 1 | 1 |
| 5. Was a sample size justification, power description, or variance and effect estimates | 0 | 0 | 0 | 0 | 0 | 0 | 0 | 0 | 0 | 0 | 0 | 0 | 0 | 0 | 0 |
| 6.For the analyses in this paper, were the exposure (s) of interest measured prior to the outcome(s) being measured? | 1 | 1 | 1 | 1 | 1 | 1 | 1 | 1 | 1 | 1 | 1 | 1 | 1 | 1 | 1 |
| 7. Was the timeframe sufficient so that one could reasonably expect to see an association between exposure and outcome if it existed? | 0 | 1 | 1 | 1 | 1 | 1 | 1 | 1 | 0 | 1 | 1 | 1 | 1 | 1 | 1 |
| 8. For exposures that can vary in amount or level, did the study examine different levels of the exposure as related to the outcome (e.g., categories of exposure, or exposure measured as continuous variable)? | 1 | 1 | 1 | 1 | 1 | 1 | 1 | 1 | 1 | 1 | 1 | 1 | 1 | 1 | 1 |
| 9. Were the exposure measures (independent variables) clearly defined, valid, reliable, and implemented consistently across all study participants? | 1 | 0 | 1 | 1 | 1 | 1 | 1 | 1 | 1 | 1 | 1 | 1 | 1 | 1 | 1 |
| 10. Was the exposure(s) assessed more than once over time? | 1 | 1 | 0 | 0 | 0 | 0 | 0 | 0 | 1 | 0 | 0 | 0 | 1 | 0 | 1 |
| 11. Were the outcome measures (dependent variables) learly defined, valid, reliable, and implemented consistently across all study participants? | 1 | 1 | 1 | 1 | 1 | 1 | 1 | 1 | 1 | 1 | 1 | 1 | 1 | 1 | 1 |
| 12. Were the outcome assessors blinded to the exposure status of participants? | * | * | * | * | * | * | * | * | * | * | * | * | * | * | * |
| 13. Was loss to follow-up after baseline 20% or less? | 1 | 1 | 1 | 1 | 1 | 1 | 1 | 1 | 1 | 1 | 1 | 1 | 1 | 1 | 1 |
| 14. Were key potential confounding variables measured and adjusted statistically for their impact on the relationship between exposure(s) and outcome(s)? | 0 | 1 | 1 | 1 | 0 | 1 | 1 | 1 | 1 | 1 | 1 | 0 | 1 | 0 | 1 |
| Total score (out of 14) | 10/14 | 11/14 | 10/14 | 10/14 | 10/14 | 11/14 | 11/14 | 11/14 | 11/14 | 11/14 | 11/14 | 10/14 | 11/14 | 10/14 | 11/14 |

|  | Lee 2002 | Lee 2011 | Li 2021 | Liang 2019 | Lim 2019 | Liu 2019 | Malzoni 2009 | Margina 2008 | Mendivil 2016 | Nam 2012 | Paik 2019 | Park 2013 | Park 2016 | Yuan 2019 |
| --- | --- | --- | --- | --- | --- | --- | --- | --- | --- | --- | --- | --- | --- | --- |
| 1.Was the research question or objective in this paper clearly stated? | 1 | 1 | 1 | 1 | 1 | 1 | 1 | 1 | 1 | 1 | 1 | 1 | 1 | 1 |
| 2.Was the study population clearly specified and defined? | 1 | 1 | 1 | 1 | 1 | 1 | 1 | 1 | 1 | 1 | 1 | 1 | 1 | 1 |
| 3.Was the participation rate of eligible persons at least 50%? | * | 1 | 1 | 1 | 1 | 1 | 1 | 1 | 1 | * | 1 | 1 | 1 | 1 |
| 4.Were all the subjects selected or recruited from the same or similar populations (including the same time period)? Were inclusion and exclusion criteria for being in the study prespecified and applied uniformly to all participants? | 0 | 1 | 1 | 1 | 1 | 1 | 1 | 1 | 1 | 0 | 1 | 1 | 1 | 1 |
| 5. Was a sample size justification, power description, or variance and effect estimates | 0 | 0 | 0 | 0 | 0 | 0 | 0 | 0 | 0 | 0 | 0 | 0 | 0 | 0 |
| 6.For the analyses in this paper, were the exposure (s) of interest measured prior to the outcome(s) being measured? | 1 | 1 | 1 | 1 | 1 | 1 | 1 | 1 | 1 | * | 1 | 1 | 1 | 1 |
| 7. Was the timeframe sufficient so that one could reasonably expect to see an association between exposure and outcome if it existed? | * | 1 | 1 | 1 | 1 | 1 | 1 | 1 | 1 | 1 | 1 | 1 | 1 | 1 |
| 8. For exposures that can vary in amount or level, did the study examine different levels of the exposure as related to the outcome (e.g., categories of exposure, or exposure measured as continuous variable)? | 1 | 1 | 1 | 1 | 1 | 1 | 1 | 1 | 1 | 0 | 1 | 1 | 1 | 1 |
| 9. Were the exposure measures (independent variables) clearly defined, valid, reliable, and implemented consistently across all study participants? | 1 | 1 | 1 | 1 | 1 | 1 | 1 | 1 | 1 | 1 | 1 | 1 | 1 | 1 |
| 10. Was the exposure(s) assessed more than once over time? | 0 | 0 | 0 | 0 | 0 | 0 | 0 | 0 | 0 | 0 | 0 | 0 | 0 | 0 |
| 11. Were the outcome measures (dependent variables) learly defined, valid, reliable, and implemented consistently across all study participants? | 1 | 1 | 1 | 1 | 1 | 1 | 1 | 1 | 1 | 1 | 1 | 1 | 1 | 1 |
| 12. Were the outcome assessors blinded to the exposure status of participants? | * | * | * | * | * | * | * | * | * | * | * | * | * | * |
| 13. Was loss to follow-up after baseline 20% or less? | 1 | 1 | 1 | 1 | 1 | 1 | 1 | 1 | 1 | 1 | 1 | 1 | 1 | 1 |
| 14. Were key potential confounding variables measured and adjusted statistically for their impact on the relationship between exposure(s) and outcome(s)? | 1 | 1 | 1 | 1 | 1 | 1 | 1 | 1 | 1 | 1 | 1 | 1 | 1 | 1 |
| Total score (out of 14) | 9/14 | 11/4 | 11/4 | 11/4 | 11/4 | 11/14 | 11/14 | 11/14 | 11/14 | 8/14 | 11/14 | 11/14 | 11/14 | 11/14 |

|  | Qin 2020 | Rodriguez 2021 | Sert 2011 | Shanmugam 2020 | Sharma 2006 | Soliman 2011 | Steed 2004 | Suh 2015 | Taylor 2011 | Topatas 2014 | Wang 2019 | Wright 2012 | Xiao 2015 | Xiao 2016 |
| --- | --- | --- | --- | --- | --- | --- | --- | --- | --- | --- | --- | --- | --- | --- |
| 1.Was the research question or objective in this paper clearly stated? | 1 | 1 | 1 | 1 | 1 | 1 | 1 | 1 | 1 | 1 | 1 | 1 | 1 | 1 |
| 2.Was the study population clearly specified and defined? | 1 | 1 | 1 | 1 | 1 | 1 | 1 | 1 | 1 | 1 | 1 | 1 | 1 | 1 |
| 3.Was the participation rate of eligible persons at least 50%? | 1 | 1 | 1 | 1 | 1 | 1 | 1 | 0 | 1 | 1 | 1 | 1 | 1 | 1 |
| 4.Were all the subjects selected or recruited from the same or similar populations (including the same time period)? Were inclusion and exclusion criteria for being in the study prespecified and applied uniformly to all participants? | 1 | 1 | 1 | 1 | 1 | 1 | 1 | * | 1 | 1 | 1 | 0 | 1 | 1 |
| 5. Was a sample size justification, power description, or variance and effect estimates | 0 | 0 | 0 | 0 | 0 | 0 | 0 | 0 | 0 | 0 | 0 | 0 | 0 | 0 |
| 6.For the analyses in this paper, were the exposure (s) of interest measured prior to the outcome(s) being measured? | 1 | 1 | 1 | 1 | 1 | 1 | 1 | 1 | 1 | 1 | 1 | 1 | 1 | 1 |
| 7. Was the timeframe sufficient so that one could reasonably expect to see an association between exposure and outcome if it existed? | 1 | 1 | 1 | 1 | 1 | 1 | 1 | 1 | 1 | 1 | 1 | 1 | 1 | 1 |
| 8. For exposures that can vary in amount or level, did the study examine different levels of the exposure as related to the outcome (e.g., categories of exposure, or exposure measured as continuous variable)? | 1 | 1 | 1 | 1 | 1 | 1 | 1 | 1 | 1 | 1 | 1 | 1 | 1 | 1 |
| 9. Were the exposure measures (independent variables) clearly defined, valid, reliable, and implemented consistently across all study participants? | 1 | 1 | 1 | 1 | 1 | 1 | 1 | 1 | 1 | 1 | 1 | 1 | 1 | 1 |
| 10. Was the exposure(s) assessed more than once over time? | 0 | 0 | 0 | 0 | 0 | 0 | 0 | 0 | 0 | 0 | 0 | 0 | 0 | 0 |
| 11. Were the outcome measures (dependent variables) learly defined, valid, reliable, and implemented consistently across all study participants? | 1 | 1 | 1 | 1 | 1 | 1 | 1 | 1 | 1 | 1 | 1 | 1 | 1 | 1 |
| 12. Were the outcome assessors blinded to the exposure status of participants? | * | * | * | * | * | * | * | * | * | * | * | * | * | * |
| 13. Was loss to follow-up after baseline 20% or less? | 1 | 1 | 1 | 1 | 0 | 1 | 1 | 1 | 1 | 1 | 1 | 0 | 1 | 1 |
| 14. Were key potential confounding variables measured and adjusted statistically for their impact on the relationship between exposure(s) and outcome(s)? | 1 | 1 | 1 | 1 | 1 | 1 | 1 | 1 | 1 | 1 | 1 | 1 | 1 | 1 |
| Total score (out of 14) | 11/14 | 11/14 | 11/14 | 11/14 | 10/14 | 11/14 | 11/14 | 8/14 | 11/14 | 11/14 | 11/14 | 9/14 | 11/14 | 11/14 |

Key: 1 = Yes, 0 = No, * = Not reported, N/A = Not applicable.
