# Supplementary material for: Meta-analysis of laparoscopic radical hysterectomy, excluding robotic assisted versus open radical hysterectomy for early stage cervical cancer
Source: Sci Rep. 2023 Jan 6;13:273. doi: 10.1038/s41598-023-27430-9 (PMC9822966; doi:10.1038/s41598-023-27430-9)
Supplement: Supplementary file 3 — Supplementary Information 3. [file 41598_2023_27430_MOESM3_ESM.docx]

**1. Operative time (minutes)**

**
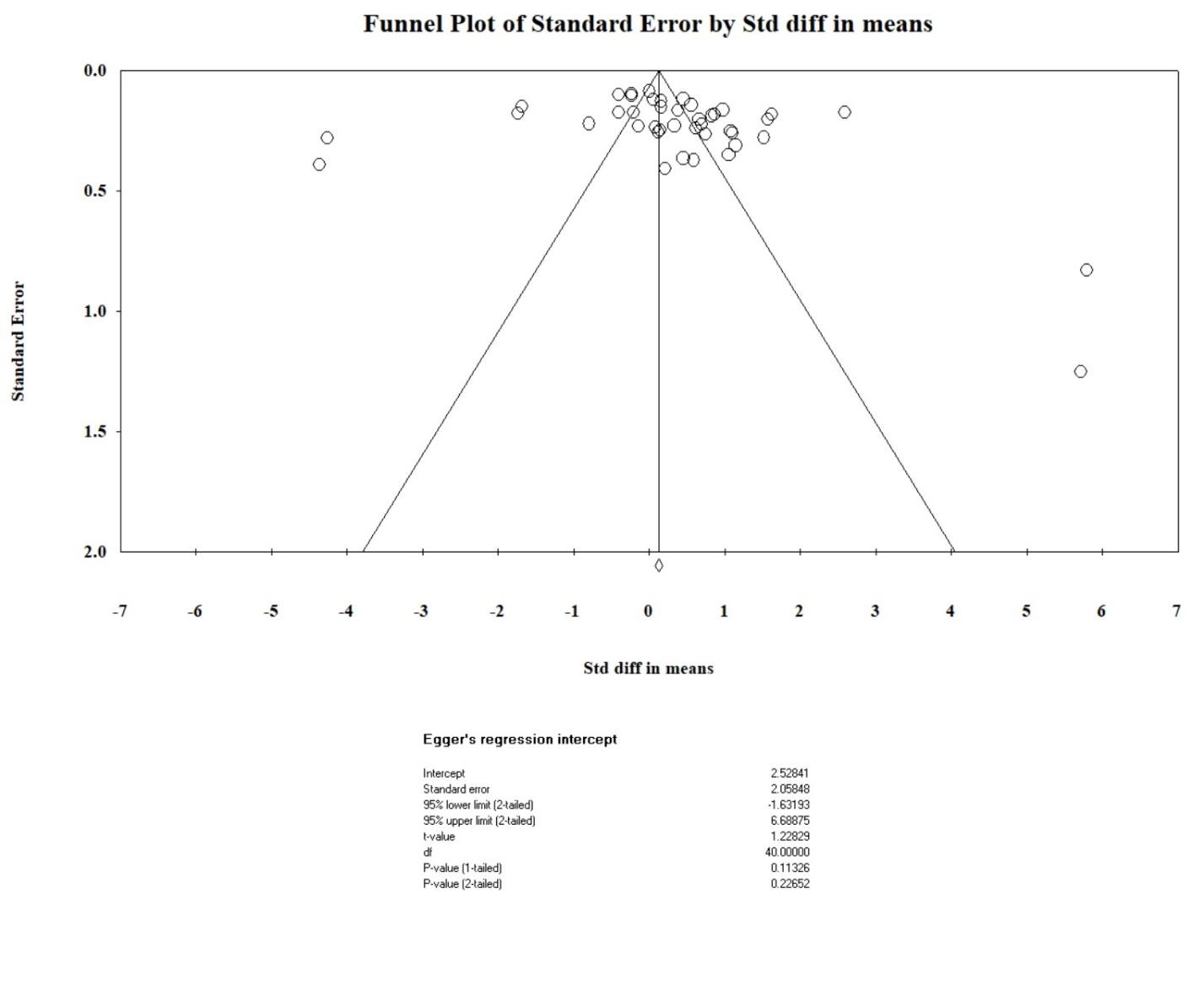
**

**2. Estimated blood loss (ml)**

**
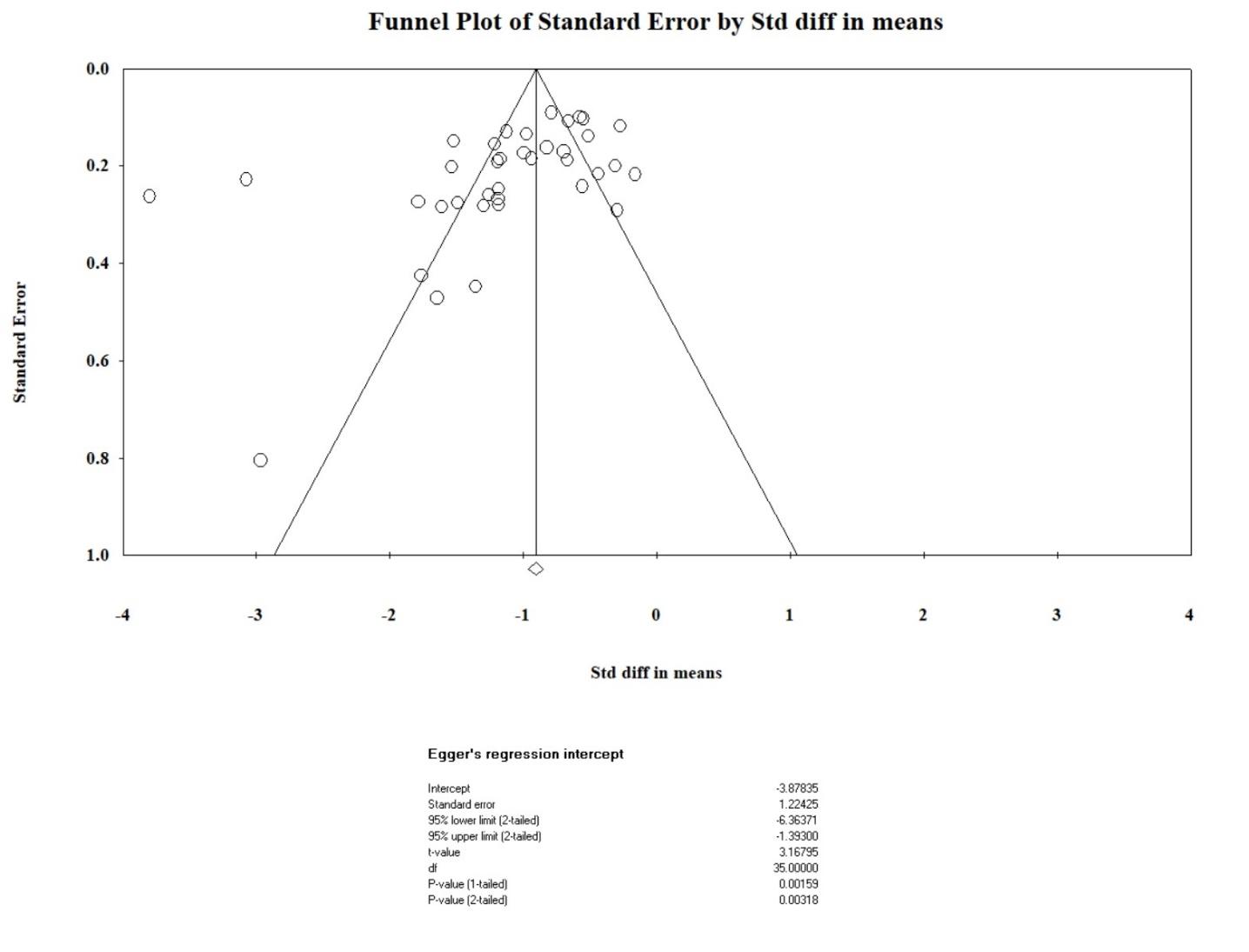
**

**3. Intraoperative complication**

**
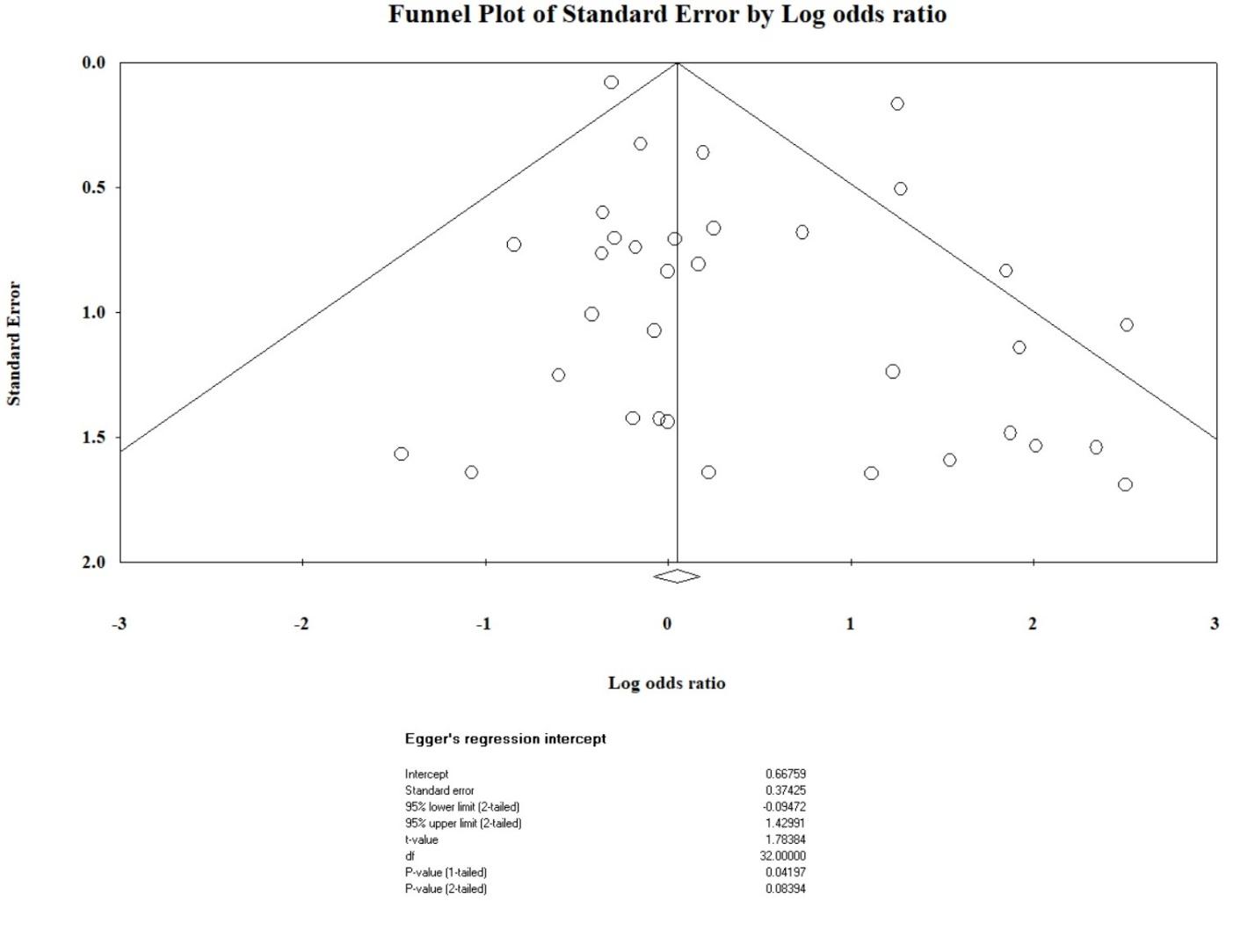
**

**4. Postoperative complication**


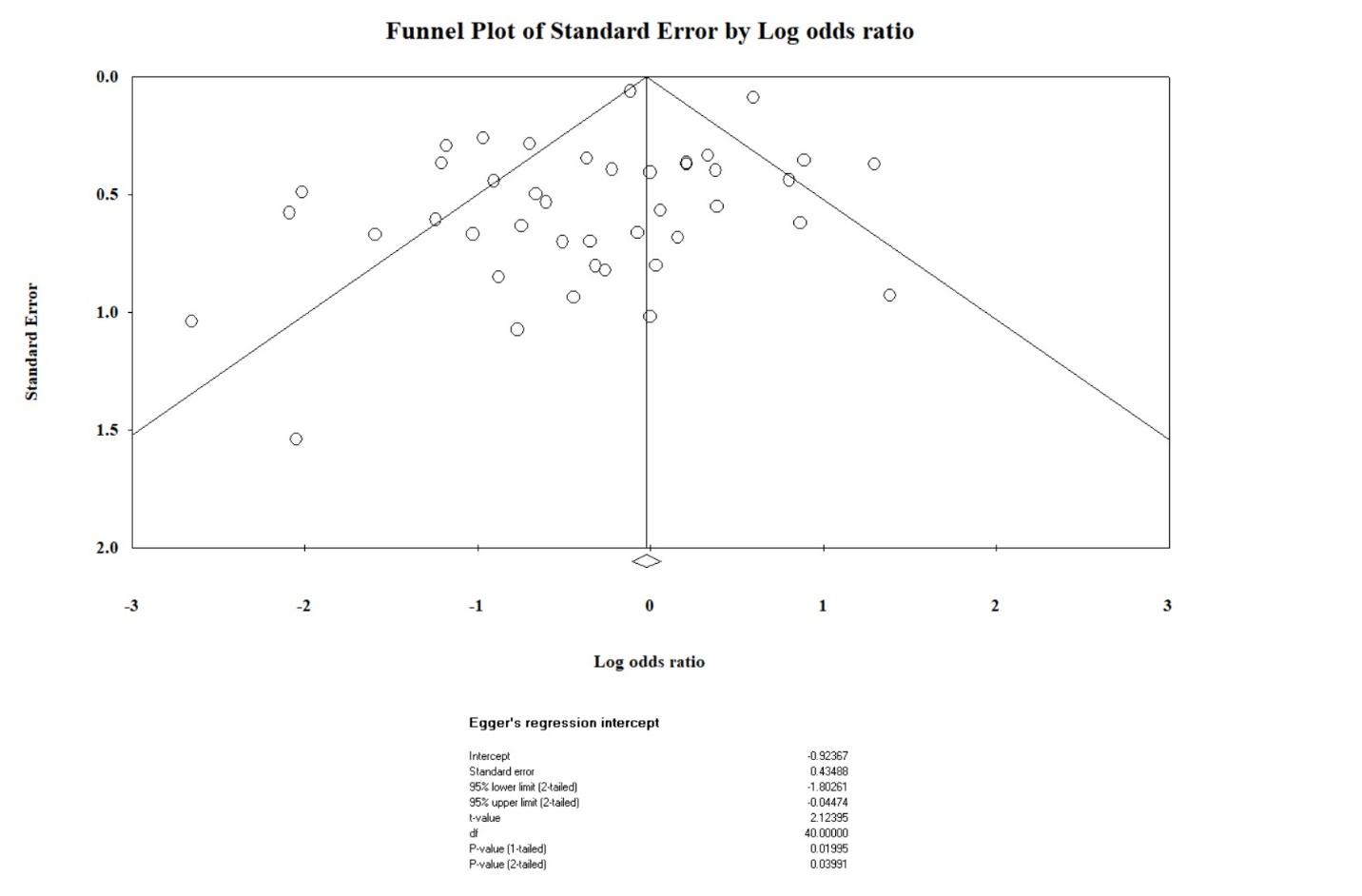


**5. Length of hospital stay (days)**


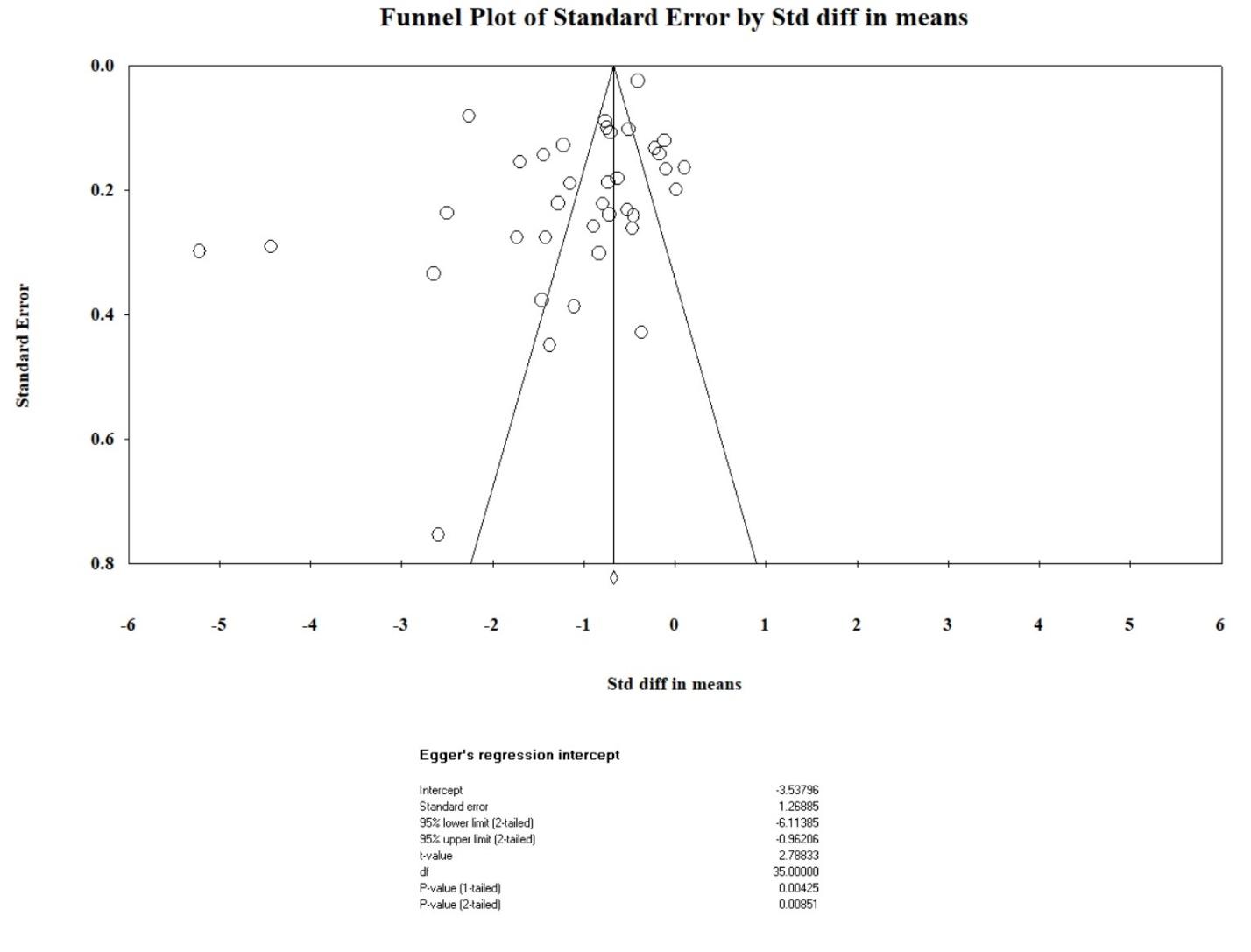


**6. Resected lymph nodes**


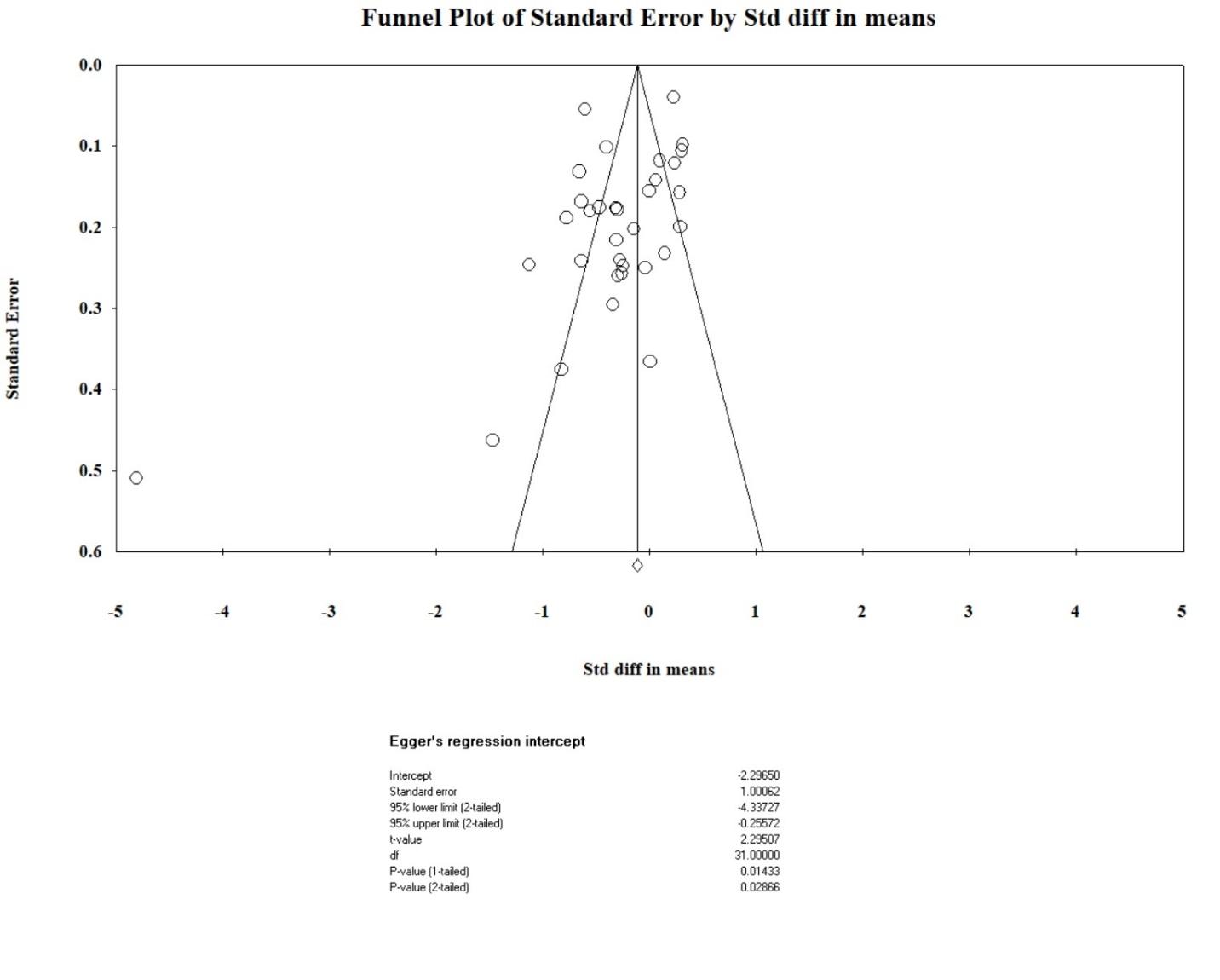


**7. Five-year Overall Survival**


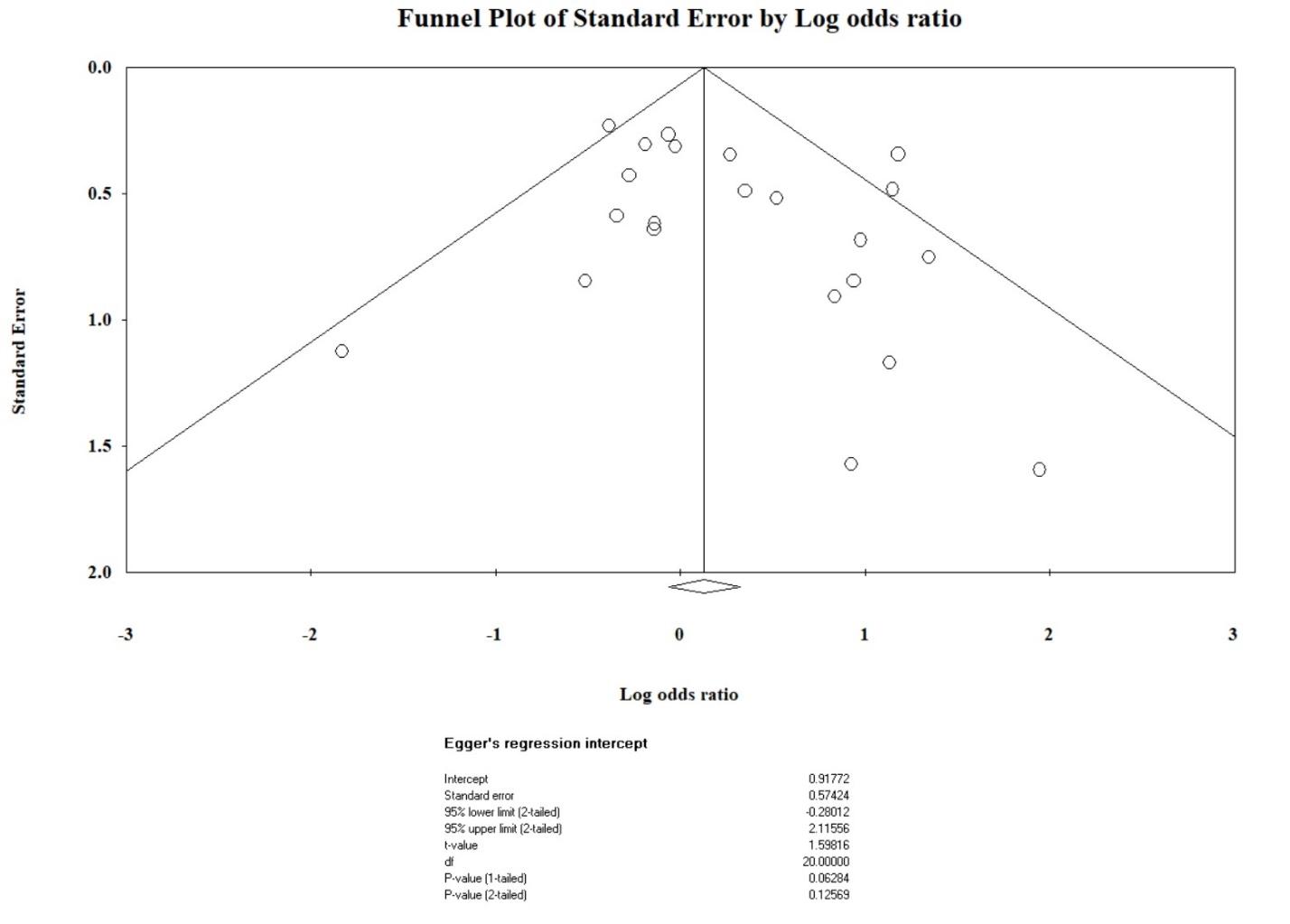


**8. Disease free survival**


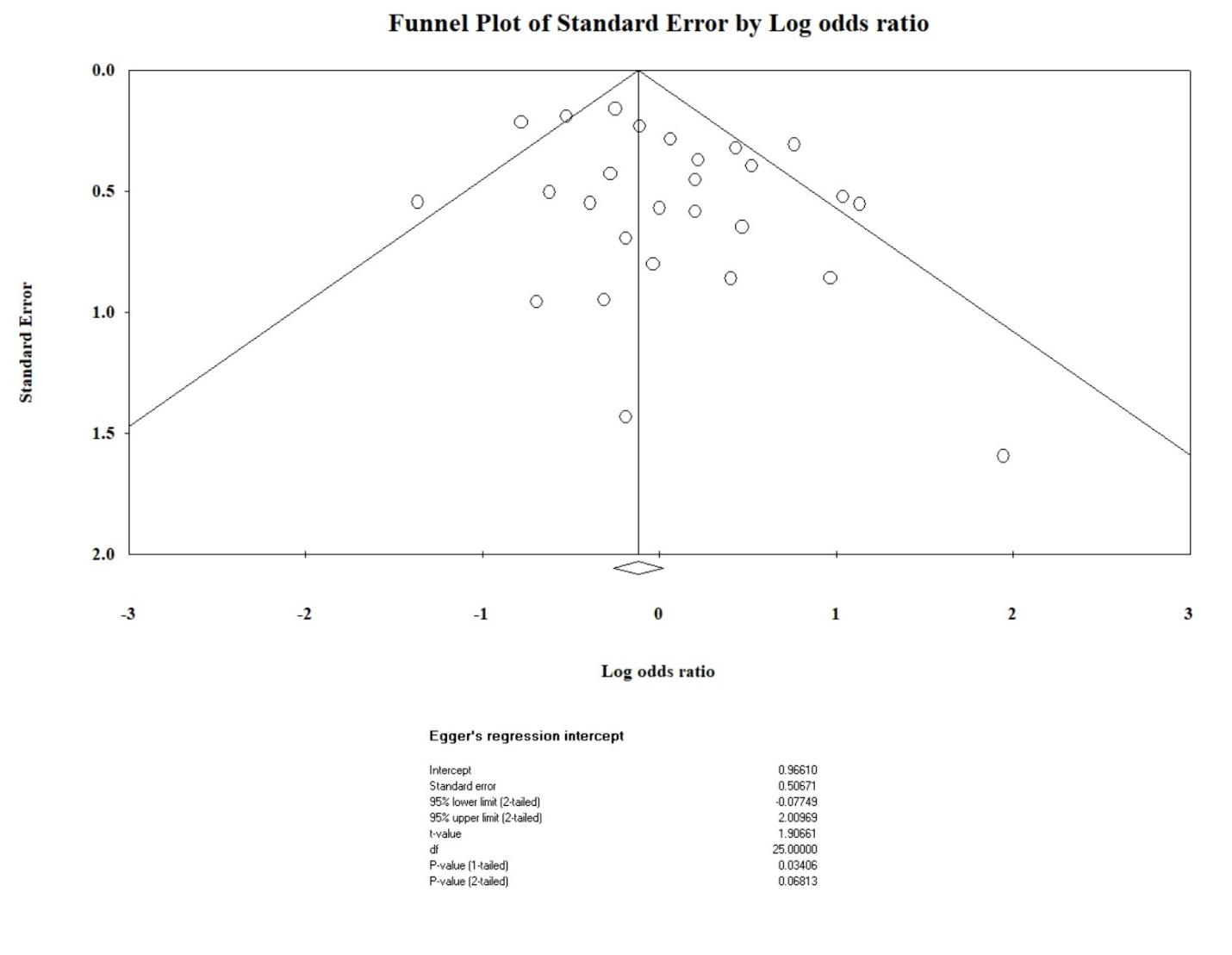


**9. Mortality
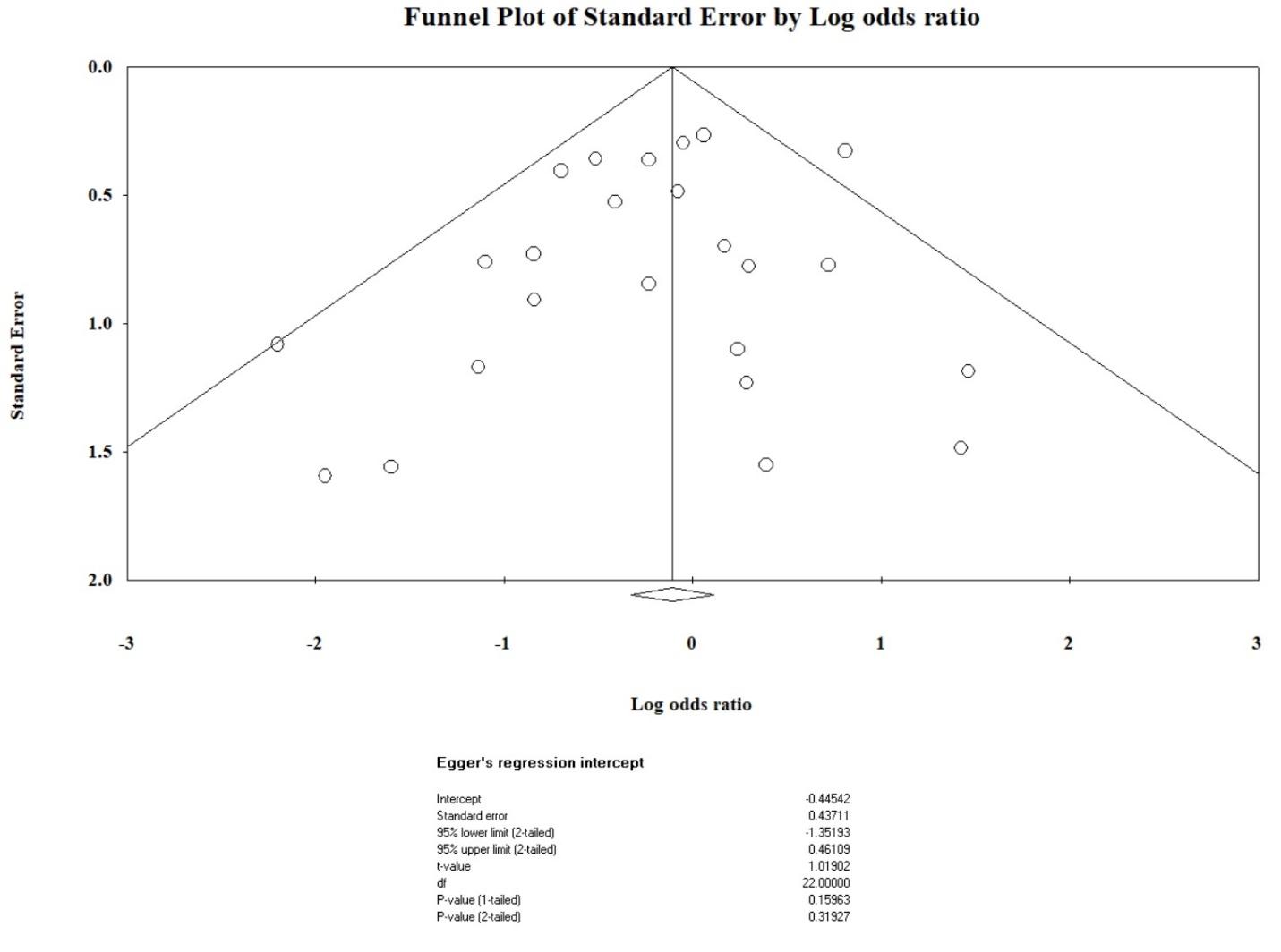
**

**10. Recurrence**

**
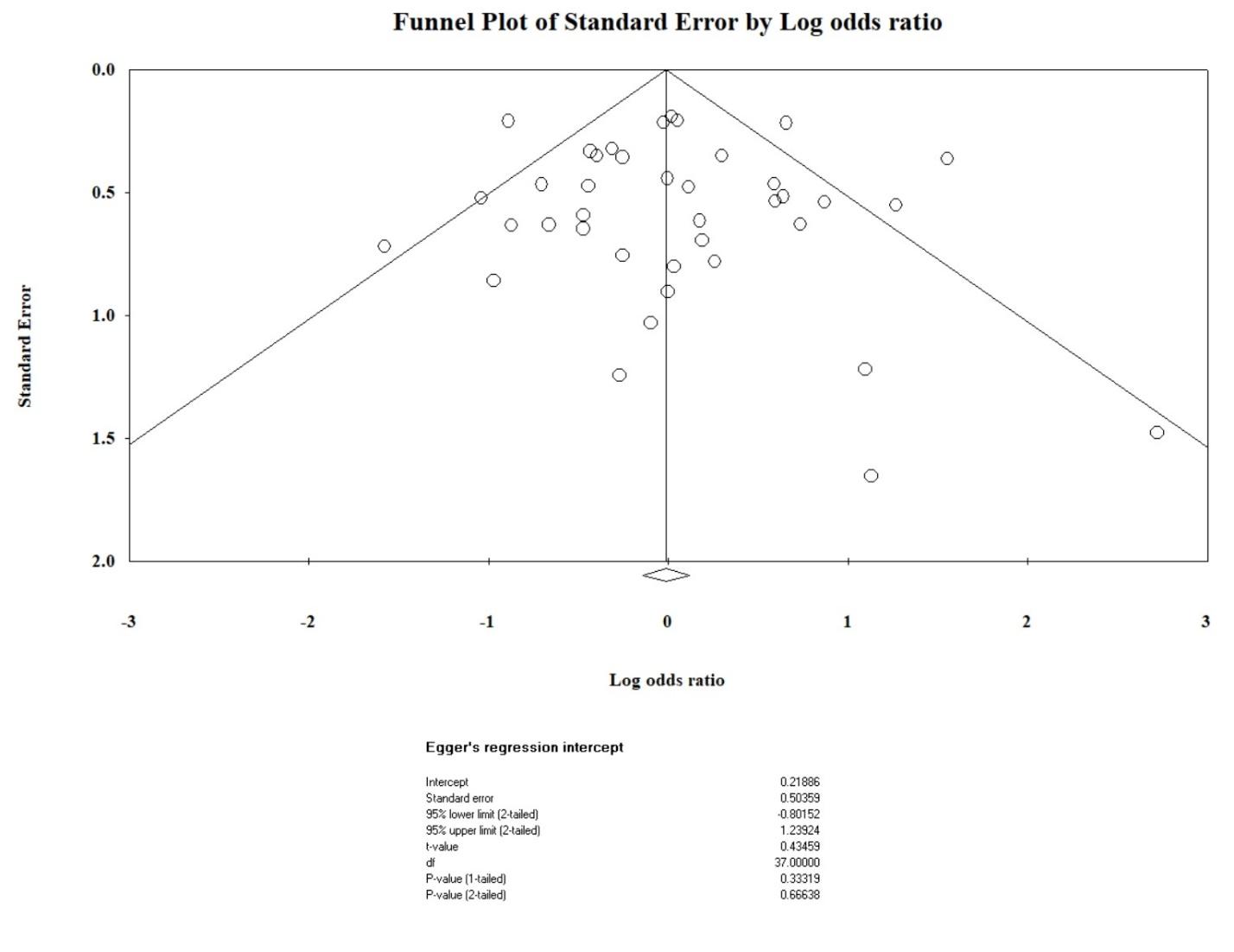
**

**11. Blood transfusion rate**

**
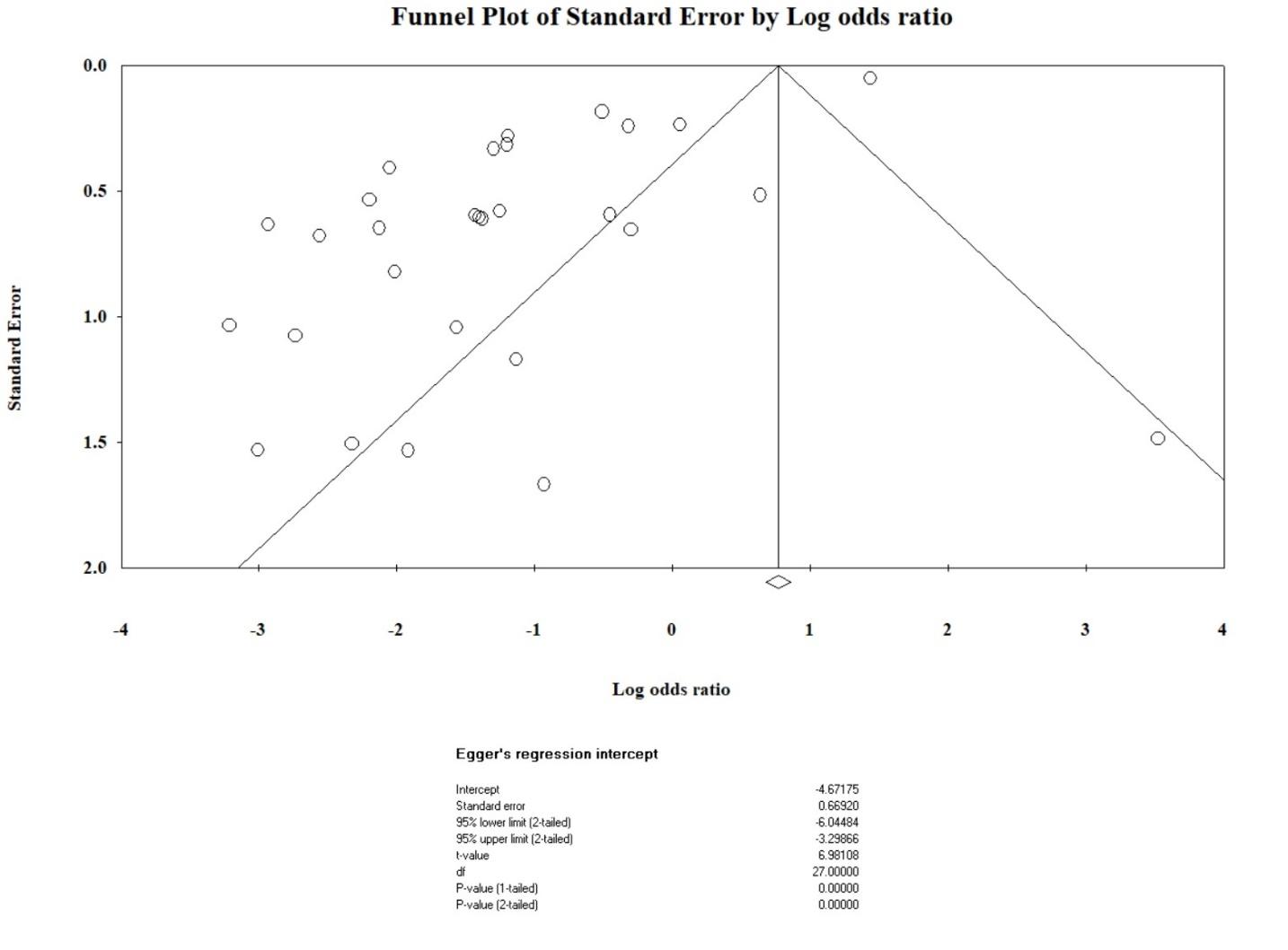
**
