# Supplementary material for: Meta-analysis of laparoscopic radical hysterectomy, excluding robotic assisted versus open radical hysterectomy for early stage cervical cancer
Source: Sci Rep. 2023 Jan 6;13:273. doi: 10.1038/s41598-023-27430-9 (PMC9822966; doi:10.1038/s41598-023-27430-9)

1. **Operative time (minutes)**


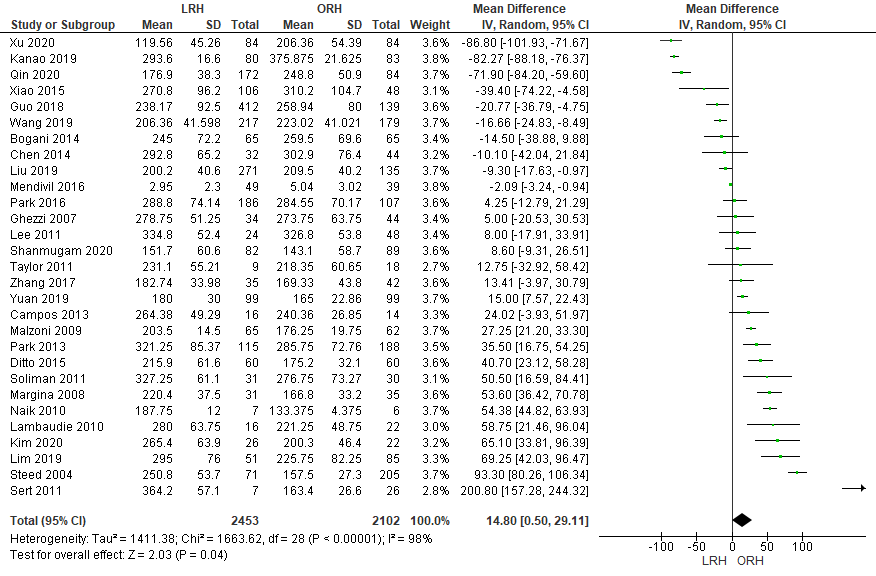


1. **Estimated blood loss (ml)**


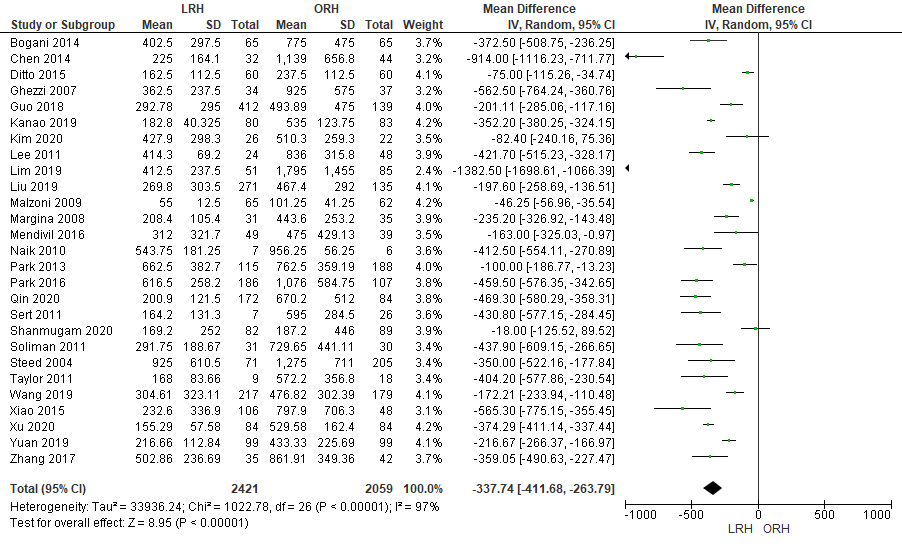


1. **Intraoperative complication**


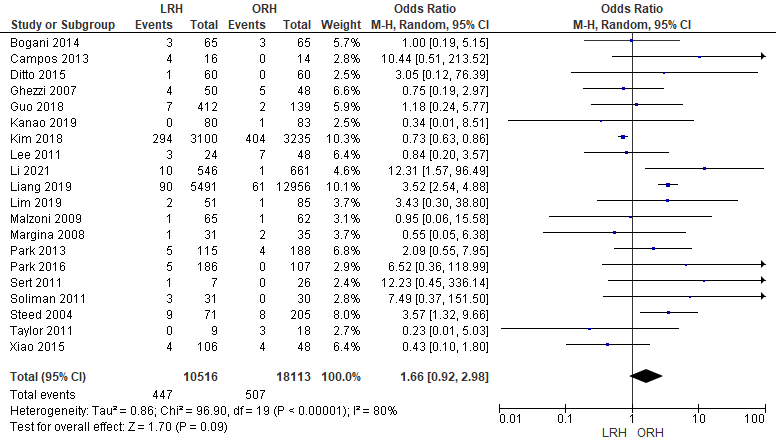


1. **Postoperative complication**
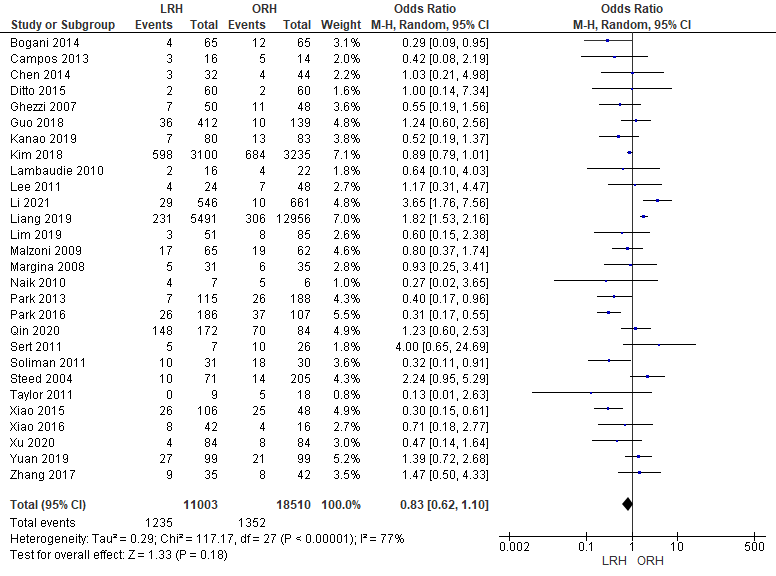

2. **Length of hospital stay (days)**


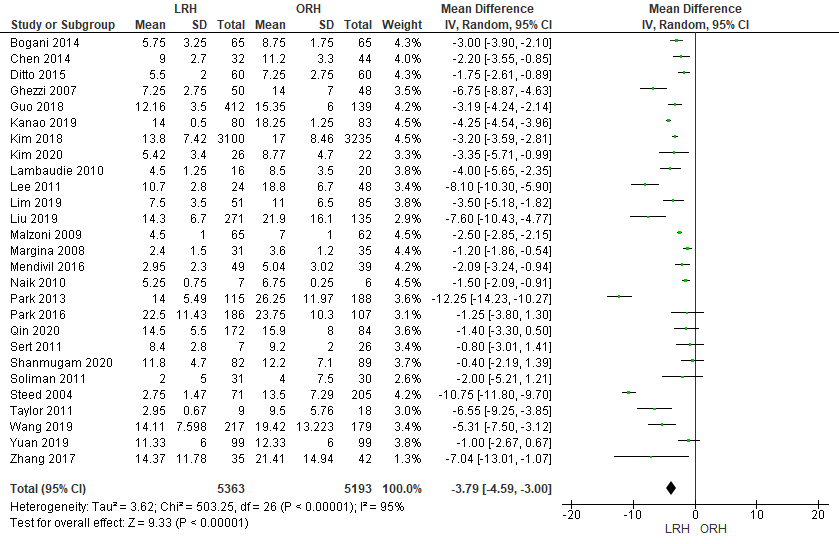


1. **Resected lymph nodes**


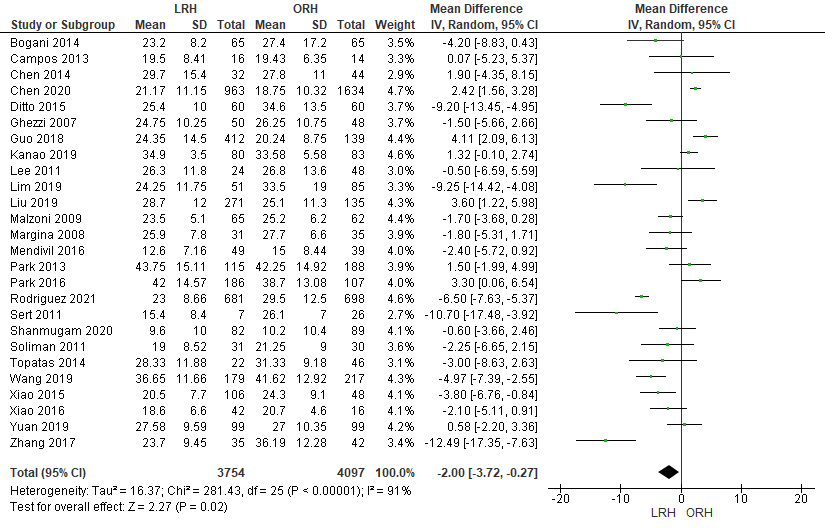


1. **Five-year Overall Survival**


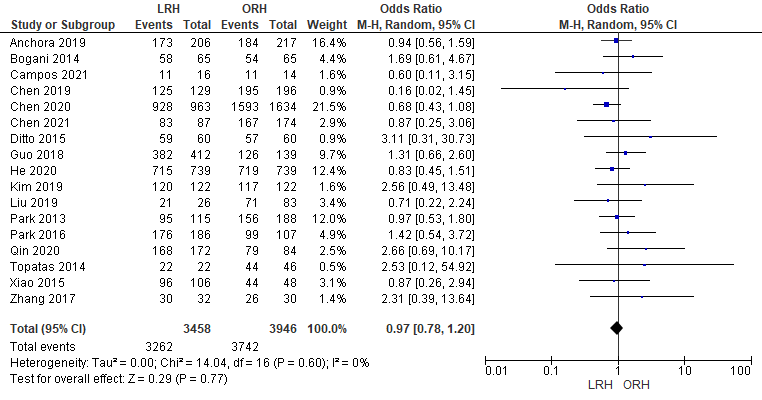


1. **Disease free survival**


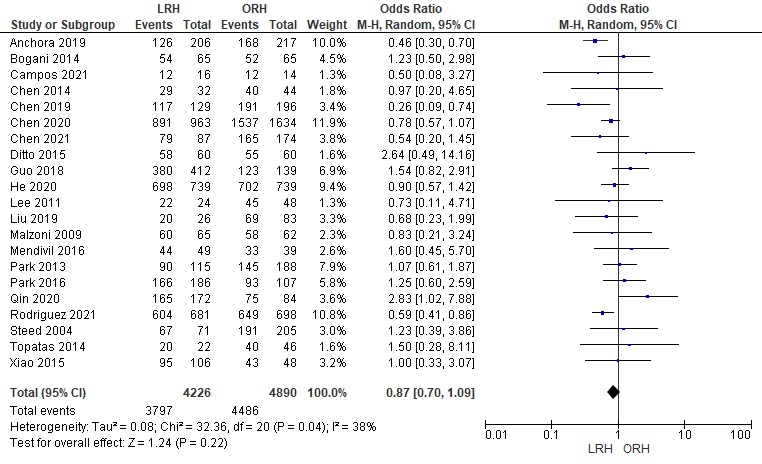


1. **Mortality**


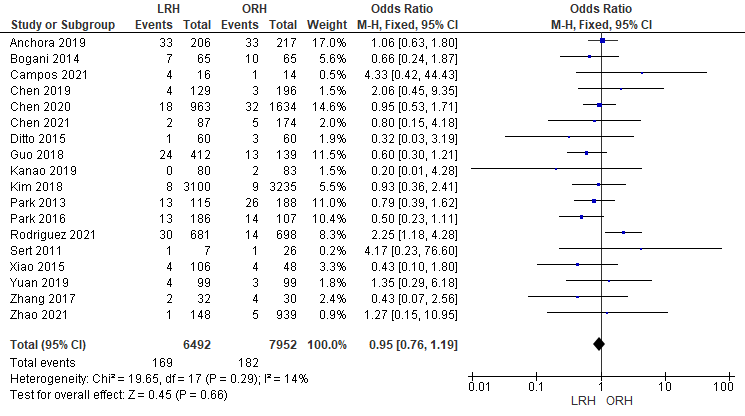


1. **Recurrence**


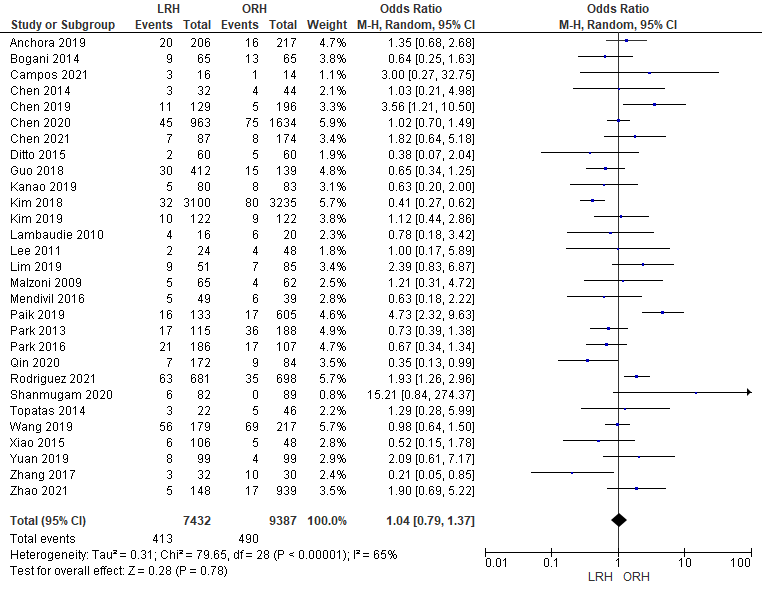


1. **Blood transfusion rate**


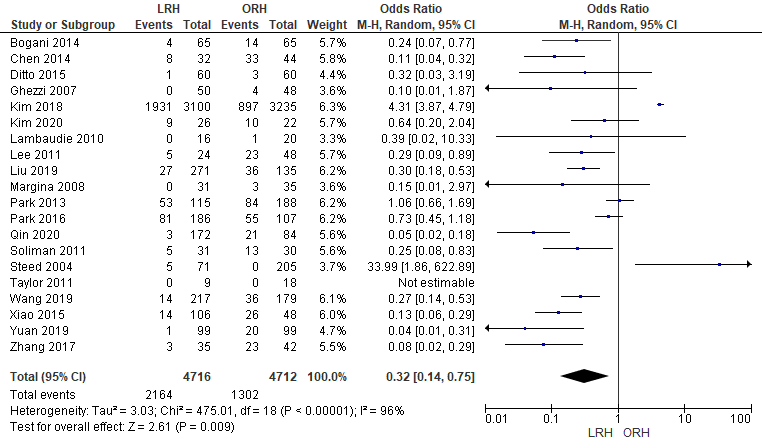

Supplement: Supplementary file 4 — Supplementary Information 4. [file 41598_2023_27430_MOESM4_ESM.docx]
