# Supplementary material for: Meta-analysis of laparoscopic radical hysterectomy, excluding robotic assisted versus open radical hysterectomy for early stage cervical cancer
Source: Sci Rep. 2023 Jan 6;13:273. doi: 10.1038/s41598-023-27430-9 (PMC9822966; doi:10.1038/s41598-023-27430-9)
Supplement: Supplementary file 5 — Supplementary Legends. [file 41598_2023_27430_MOESM5_ESM.docx]

**Supplementary Table Legend:**

Supplementary Table S1: GRADE quality assessments for included studies.

Supplementary Table S2: Quality assessment for included retrospective studies

**Supplementary Figure Legend:**

Supplementary File S1: Funnel plot and Egger test of all outcomes.

Supplementary File S2: Sensitivity analysis of all studies with a low risk of bias.
